# Yield trends, variability and stagnation analysis of major crops in France over more than a century

**DOI:** 10.1038/s41598-018-35351-1

**Published:** 2018-11-15

**Authors:** Bernhard Schauberger, Tamara Ben-Ari, David Makowski, Tomomichi Kato, Hiromi Kato, Philippe Ciais

**Affiliations:** 1 Potsdam Institute for Climate Impact Research (PIK), 14473 Potsdam, Germany; 2 Laboratoire des Sciences du Climat et de l’Environnement, Institut Pierre-Simon Laplace (IPSL), 91191 Gif sur Yvette, France; 3 INRA, AgroParisTech, UMR 211 Agronomie, Université Paris-Saclay, 78850 Thiverval-Grignon, France; 4 CIRED - Centre international de recherche sur l’environnement et le développement, UMR 8568, Nogent-sur-Marne, France; 5 Research Faculty of Agriculture, Hokkaido University, 060-8589 Sapporo, Japan; 6 Global Station for Food, Land and Water Resources, Global Institution for Collaborative Research and Education (GI-CoRE), Hokkaido University, 060-0815 Sapporo, Japan

## Abstract

France is a major crop producer, with a production share of approx. 20% within the European Union. Yet, a discussion has recently started whether French yields are stagnating. While for wheat previous results are unanimously pointing to recent stagnation, there is contradictory evidence for maize and few to no results for other crops. Here we analyse a data set with more than 120,000 yield observations from 1900 to 2016 for ten crops (barley, durum and soft wheat, maize, oats, potatoes, rapeseed, sugar beet, sunflower and wine) in the 96 mainland French *départements* (NUTS3 administrative division). We dissect the evolution of yield trends over time and space, analyse yield variation and evaluate whether growth of yields has stalled in recent years. Yields have, on average across crops, multiplied four-fold over the course of the 20^th^ century. While absolute yield variability has increased, the variation relative to the mean has halved – mean yields have increased faster than their variability. But growth of yields has stagnated since the 1990’s for winter wheat, barley, oats, durum wheat, sunflower and wine on at least 25% of their areas. Reaching yield potentials is unlikely as a cause for stagnation. Maize, in contrast, shows no evidence for stagnation.

## Introduction

Foresight studies on food availability require an accurate description of yield trends and inter-annual variability, in particular for main producers^1–3^. France is a major exporter of agricultural and food products, ranked 6^th^ in the world in 2016 according to total wheat production^4^, and also a key player in the agricultural trade market. France produced 5%, 2%, 8%, 14%, 4% and 8% of the global production of wheat, maize, barley, sugar beet, sunflower and rapeseed in 2014, respectively^4^. There have been previous assessments of the development of French agricultural productivity over time. Brisson, *et al*.^5^ analysed wheat yield trends in selected *départements* (“departments” henceforth, an administrative division in France on level 3 of the unified NUTS territory classification, NUTS3) and field trials, finding an inflection point from wheat yield growth to stagnation in 1996. Based on linear models, Calderini and Slafer^6^ found no substantial yield gains for wheat between 1900 and 1950, then strong growth until 1990, and no further yield increases since then. Ray, *et al*.^7^, using departmental yield data from 1961 to 2008, detected wheat yields as recently stagnating in 80% of the crop area. Contrary for maize, yields were found as moderately to rapidly increasing. Lin and Huybers^8^ confirmed stagnation of wheat yields around 1996, in both Northern and Southern France. Based on different types of statistical models fitted over 1950 to 2011, Michel and Makowski^9^ stated a decrease or stagnation of wheat yield growth rates from the mid-1990s (or even earlier), with few regional disparities. Grassini, *et al*.^10^ similarly used various statistical models and found that French maize and wheat yields both stalled growth in the 1990s. Analysing French maize yields at the national level from 1961 to 2010, Hawkins, *et al*.^11^ detected a slowing of technology trends towards the end of the time period, but remained inconclusive on yield stagnation. Each of these studies discusses possible driving factors for stagnation and identifies one or several of the following as relevant: lacking genetic improvement, changes in crop management (e.g., the reliance on monoculture) or legislative limitations on fertilizer use. Some of these studies also assessed changes in yield inter-annual variability. Calderini and Slafer^6^, for example, detected a mostly increasing relative stability for wheat in France, i.e. the increase in standard deviation was more than compensated by strongly increasing yields. Osborne and Wheeler^12^ found that wheat and maize variance had declined between 1961 and 2010. Iizumi and Ramankutty^13^ detected an increase of absolute wheat yield variability in major French producing areas, but a decreasing variability for maize. These elements reveal a consensus on the stagnation of wheat yield growth in France at the end of the 20^th^ century, but contrasted findings for maize. Results on yield inter-annual variability are ambiguous for wheat, but suggest a clear decline for maize. To the best of our knowledge, maize and wheat are the only crop species in France for which these questions have been studied.

We here present an in-depth analysis of the evolution of agricultural performance in France and its stability over more than a century (1900–2016) on sub-national level for ten crop species (barley, durum and soft wheat, maize, oats, potatoes, rapeseed, sugar beet, sunflower and wine). We focus on three interrelated research questions: (i) what have been the trends of major crop yields in France since 1900? In particular, have yields stagnated in recent years? (ii) what are the levels of yield variability, and how are yield trends and yield variation related? In particular, are positive yield trends associated with an increase of yield variance? (iii) is there a spatial clustering of departments for yield trends that would reveal within-country differences and help identify mechanisms?

## Results

### Yield trends and growth rates

**Figure 1:**
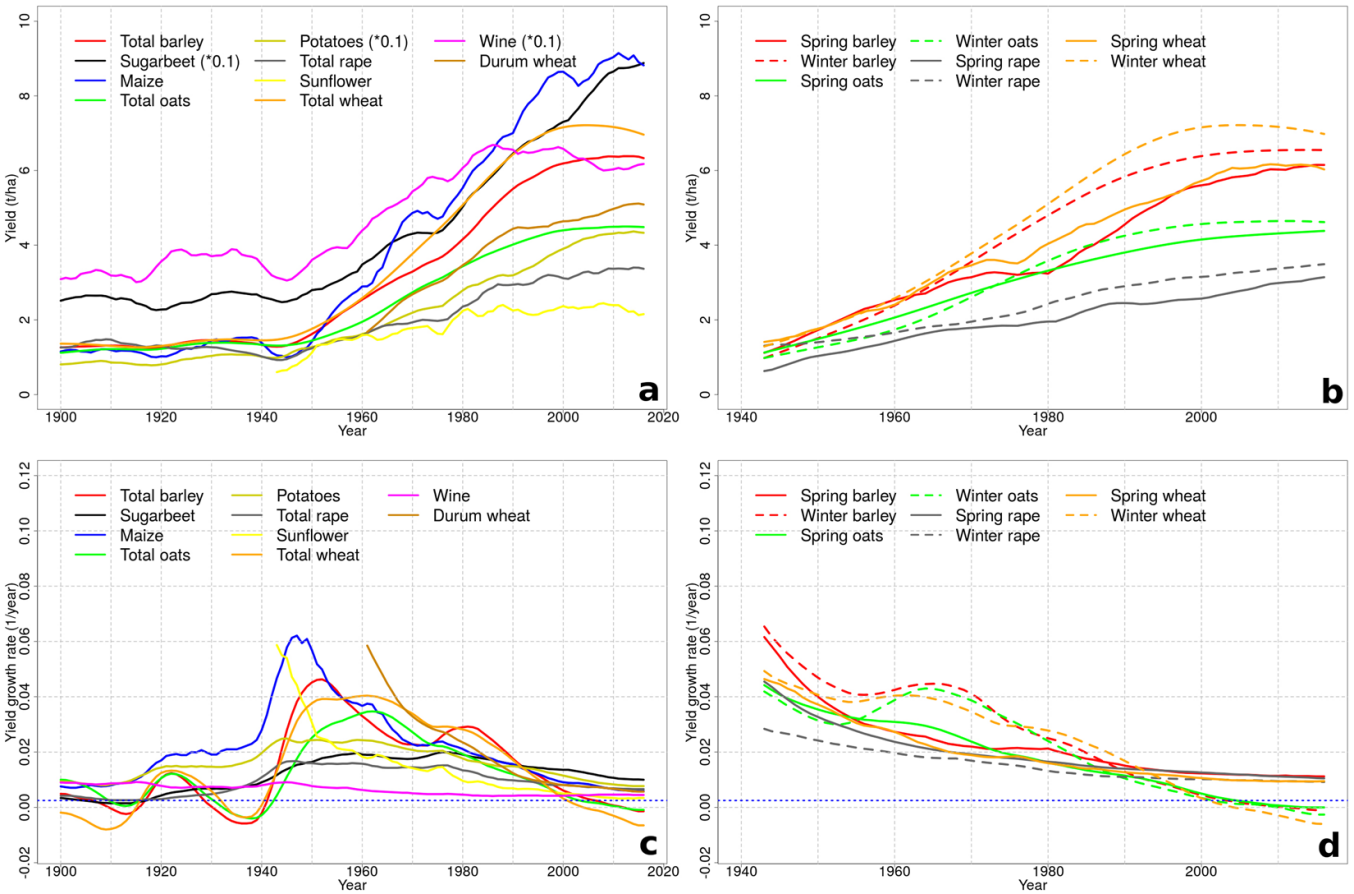
Trends and growth rates for national yields of staple French crops in the 20^th^ and 21^st^ century. (**a**) Yield trends, for one-season and season-aggregated crop species (1900–2016). (**b**) Yield trends, for spring and winter crop types (1943–2016). (**c**) Annual relative yield growth rates, for one-season and aggregated crop species. (**d**) Yield growth rates, for spring and winter crop types.(**a**,**b**) Yield trends, split by crop season types; (**c**,**d**) Annual relative yield growth rates. Some crop yields were scaled to match with the common axis range (indicated in the legend); the unit for wine is hl/ha. All values are national aggregates as area-weighted means across departments. The dashed horizontal blue lines in (**c**) and (**d**) represent the threshold below which growth rates are considered indicative for yield stagnation.

We estimated yield trends and growth rates using Dynamic Linear Models (DLM) for each crop species in each department (see Methods). DLMs allow an adjustment of trends over time and estimate annual growth rates without making strong assumption about the functional form of yield trends. Figure 1 presents the area-weighted averages of trends and growth rates at the national scale. All crops show a clear increase in mean yields (in tonnes dry matter per hectare, t/ha) from 1900 to 2016, with the period of strongest increase between 1950 and 1990 (Fig. 1a,b). Yields of winter varieties are systematically higher than those of summer varieties for the same crop. Trends are rather flat before 1950, followed by 30–40 years of strongly increasing yields. After 1990, yields show contrasted trends over crop species; a stagnation is observed for many species (in particular cereals), but not for all (e.g., sugarbeet or maize; see below). Minimum and maximum yields have increased for almost all crops across departments between 1900 and 2016 (SI Fig. 1). In recent decades, though, trends are diverging between crops, with maximum yields having stalled for oats, sunflower, durum and soft wheat and wine. Minimum yields are not further increasing for oats, potatoes, rape, sunflower and wine.

Growth rates, relative to mean yields, were positive for all crops between 1940 and 2000, but have been declining with time, that is, improvement of yields has slowed down after a peak in mid 20^th^ century. Negative growth rates occur for some crops outside the 1940–2000 window (Fig. 1c,d). In particular barley, oats, soft wheat, durum wheat and maize yields had sustained growth rates of at least 2 and up to 6% per year between 1950 and 1980. But these rates have declined after 2000 to nearly zero or even negative values for soft winter wheat, winter barley, spring and winter oats, wine and sunflower; for maize they have subsided but remain positive.

**Figure 2:**
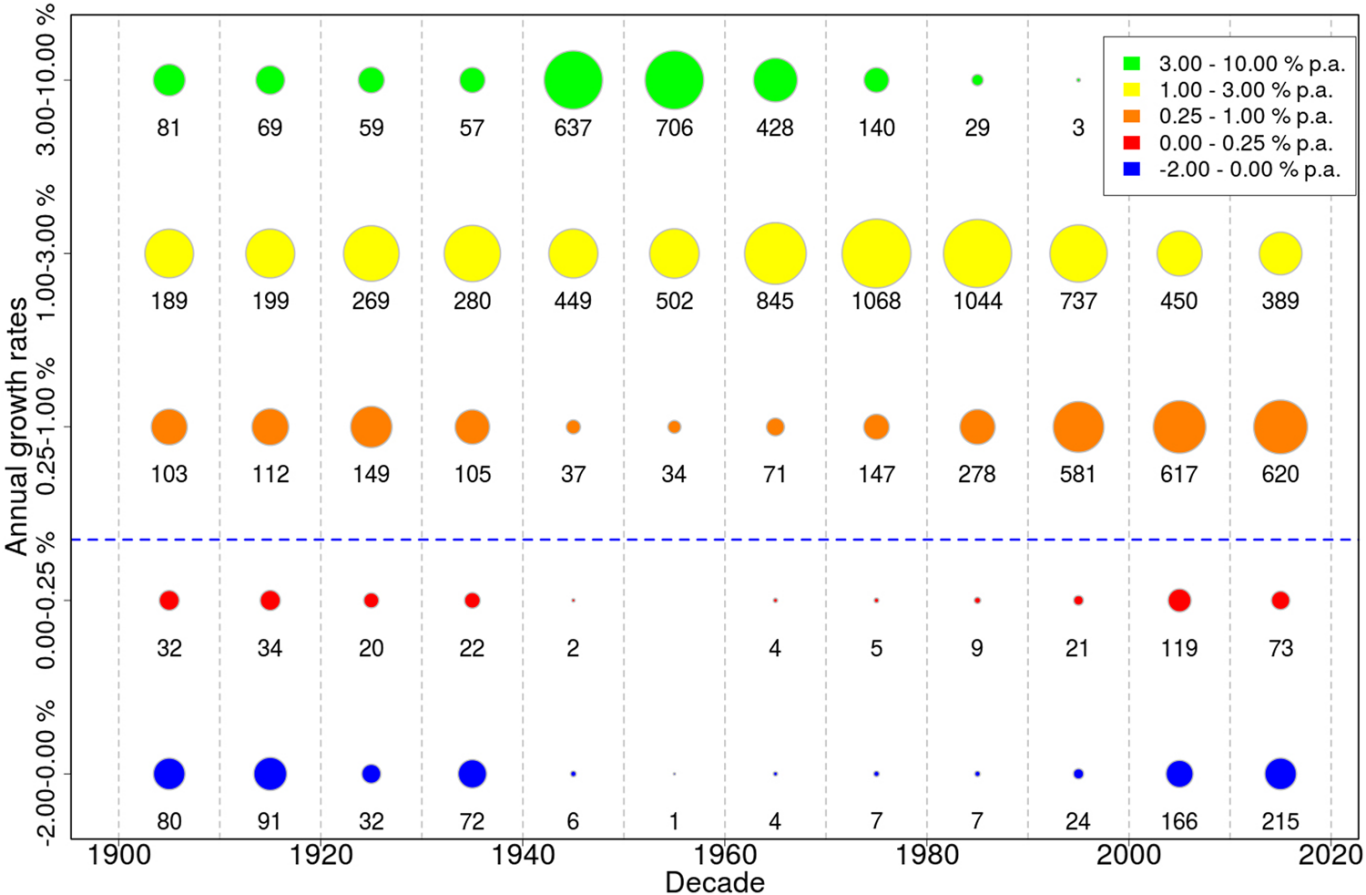
Normalized decadal relative yield growth rates over time across all crops (except wine) and departments. Growth rates are mapped to prescribed percentage categories. The area of each circle represents the number of crop-department cases that fall into this category, and the exact number of crop-department entries per bubble is indicated. The blue dashed line marks the stagnation detection threshold, i.e. a growth rate lower than this cut-off is considered indicative of stagnating or declining yields, in contrast to times of rapid growth in mid-20^th^ century (see Methods). Wine is omitted here since its growth rates are strongly subject to voluntary limitation of yields, which would blur the overall picture (see Discussion).

Decadal normalized relative yield growth rates (see Methods for categorization) for all crops and all departments are summarized in Fig. 2 and confirm the results obtained at the national scale. When all crop-department cases are considered together, yield growth rates exhibit a bell-shaped pattern over the duration of the records: slow growth at the beginning of the 20^th^ century followed by decades of rapid yield increase from the 1940s until approximately 1980, and a slowdown of this increase eventually leading to stagnation or even negative growth rates after 2000.

Yield trends are highly correlated with trends in mineral nitrogen (N) fertilizer input (average Pearson’s *r* = 0.72). This is true for all crop species considered here (SI Fig. 9a). Potassium (K_2_O) trends are also correlated with yields but the correlation is smaller (*r* = 0.34), and lacking for durum wheat. Phosphate (P_2_O_5_) application, in contrast, is not correlated with yield trends.

**Figure 3:**
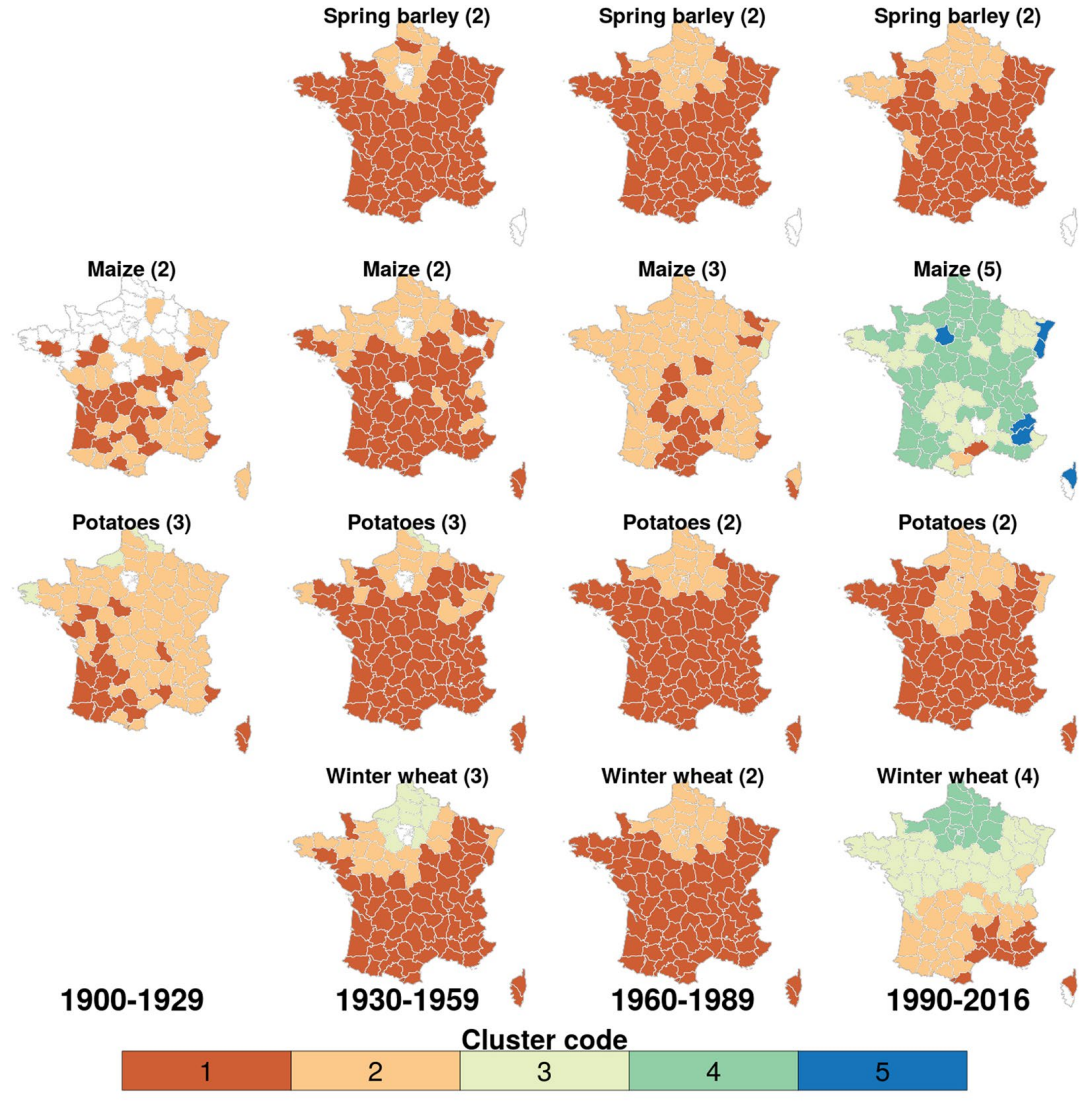
Spatial clustering of mean yields for selected crops over time. Departments are assigned to the same cluster if they show similar mean yield levels within the indicated time frames. Cluster colours reflect mean yields, with higher numbers indicating higher yields (mapping in SI Table 2). The optimal cluster number is determined separately for each crop and time period and is written in brackets after the crop name.

A spatial clustering of yield levels is apparent for several crops: winter and spring barley, all oat types, potatoes, winter and total wheat, and durum wheat (Fig. 3 for four selected crops with large cultivated areas, SI Fig. 6 for all crops). It has limitedly evolved over time, considering four consecutive periods of ca. 30 years. The number of clusters tends to increase over time, revealing an increase of the divergence of average yields between regions. No reliable spatial clustering can be identified for the other crops. Clusters with higher average yields are localized in Northern France for all crops except maize and wine. Higher yields in the North are consistent with higher fertilizer application rates in this region (SI Fig. 10). The Northern part of France is known to be composed of intensive production conditions^14^. The clusters highlight strong differences in agricultural productivity among major agricultural areas in France and reflect a widening of the productivity gap over time.

### Inter-annual yield variability

**Figure 4:**
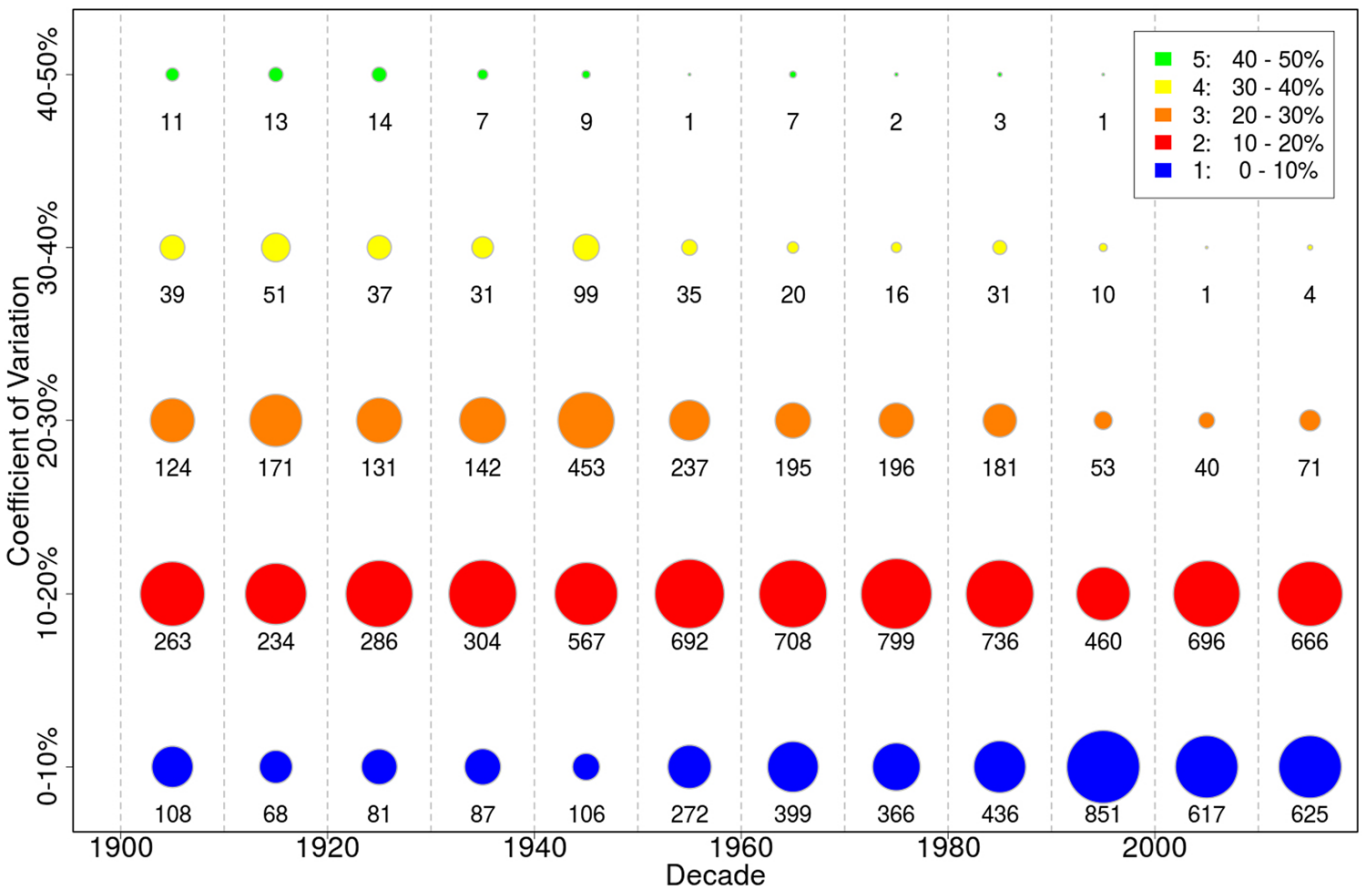
Coefficient of yield variation (CV, defined as standard deviation over mean during each decade) for all crops and departments per decade. CV was calculated separately per crop, department and decade. Areas of circles correspond to the number of data points in this decade and category (exact number is given below each circle), normalized by the number of total values per decade. Detailed values per crop are available in SI Fig. 7a.

When expressed relative to decadal mean yields, the variability of crop yields (as CV, Coefficient of Variation, defined as standard deviation over mean) shows a decreasing trend over time for a large proportion of crop-department combinations (Fig. 4; SI Fig. 7a). This is illustrated by the decreasing share of values in the categories of moderate to high CV (i.e. CV > 20%, categories 3–5 in Fig. 4), almost vanishing after mid-20^th^ century, and the increasing share of values in lower categories (CV < 20%, categories 1–2). This decline of CV is not due to a decrease of yield standard deviation over time (SI Fig. 7b). The standard deviations of yield have indeed increased over time for all crops except potatoes, winter rape and wine, but the increase rates of standard deviations were lower than those of mean yields –explaining why the CVs have decreased over time. A Taylor power plot (SI Fig. 12) conveys the same result that absolute yield variability has increased with mean yield for all crops, except potatoes, winter rape and wine. The rate of increase has declined for both mean yields and standard deviation in more recent decades, evidenced by the denser clustering of values in later years for most crops.

Supporting evidence for a decreasing relative variability is the strong increase of minimum yields since 1900, which lowers yield variation substantially since yield losses have become less frequent or less severe (SI Fig. 1), and the consequential decrease in the upper CV quantiles (SI Fig. 7a). Notwithstanding, there has been a slight increase of the upper CV quantiles between 1990 and 2010 for spring barley, maize, winter oats and durum wheat, i.e. some departments experienced a recent increase in relative variation.

Yield residuals tend to be biased towards higher negative than positive values, i.e. they are skewed to the left (mean skewness = −0.2). This indicates that large negative deviations from expected yields are more frequent than large positive deviations. Residual means after trend removal are smaller than 0.05 t/ha in 93% of all 1,424 crops and departments, and smaller than 0.66 t/ha for all cases. Yield residuals are not correlated with variation in fertilizer input (SI Fig. 9b) – correlations with nitrogen, potassium and phosphate are all below 0.15 and insignificant. Yield variation is correlated with the extent of (national, not crop-specific) irrigated area for some crops, but not in a consistent way (SI Fig. 13).

**Figure 5:**
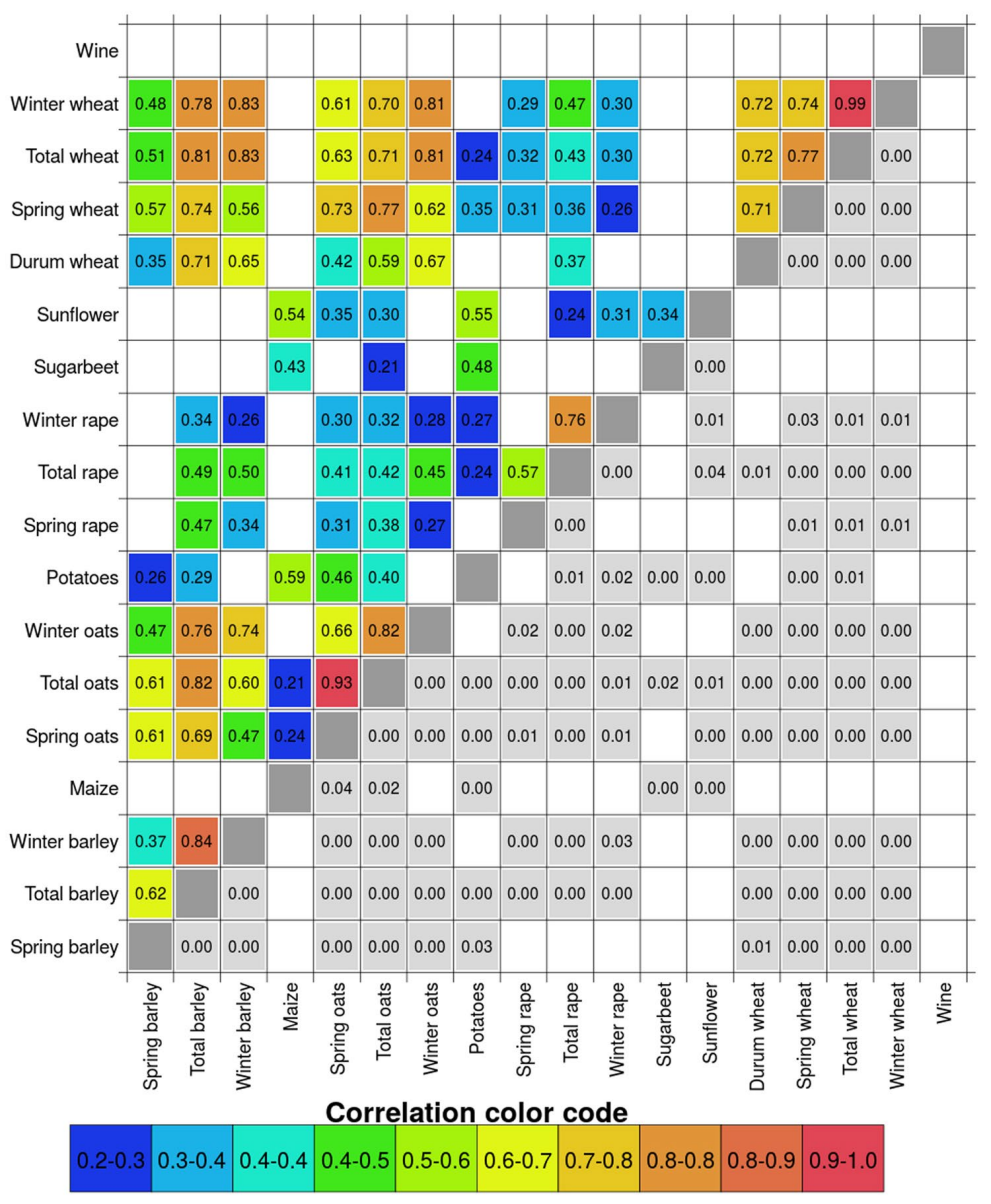
Temporal correlation (Pearson’s *r*) of yield residuals (based on nationally aggregated yield time series) between crops; only significant correlations are plotted (p < 0.05). Values above the diagonal show correlations, while numbers in grey boxes below indicate p-values. Colour coding represents correlation strength, from green = low to red = strong. For each pair of crops the maximum time frame where both time series contain data is used (SI Table 1).

The inter-annual variability of crops is correlated between crops, for average and extreme yields (Fig. 5; SI Fig. 8). Significant negative correlations between any pair of crops do not occur. Winter and spring types are co-varying for oats and wheat, but not barley and rape. The inter-annual variability of winter cereals (barley, oats and wheat) are correlated with each other above 0.6 (Pearson’s *r*) in 8 out of 9 cases (mean and extremes), indicating that similar (meteorological) conditions may be yield-determining for these crops in average and extreme yield years. Sunflower, sugar beet, maize and wine have low correlations with other crops.

### Yield stagnation

**Figure 6:**
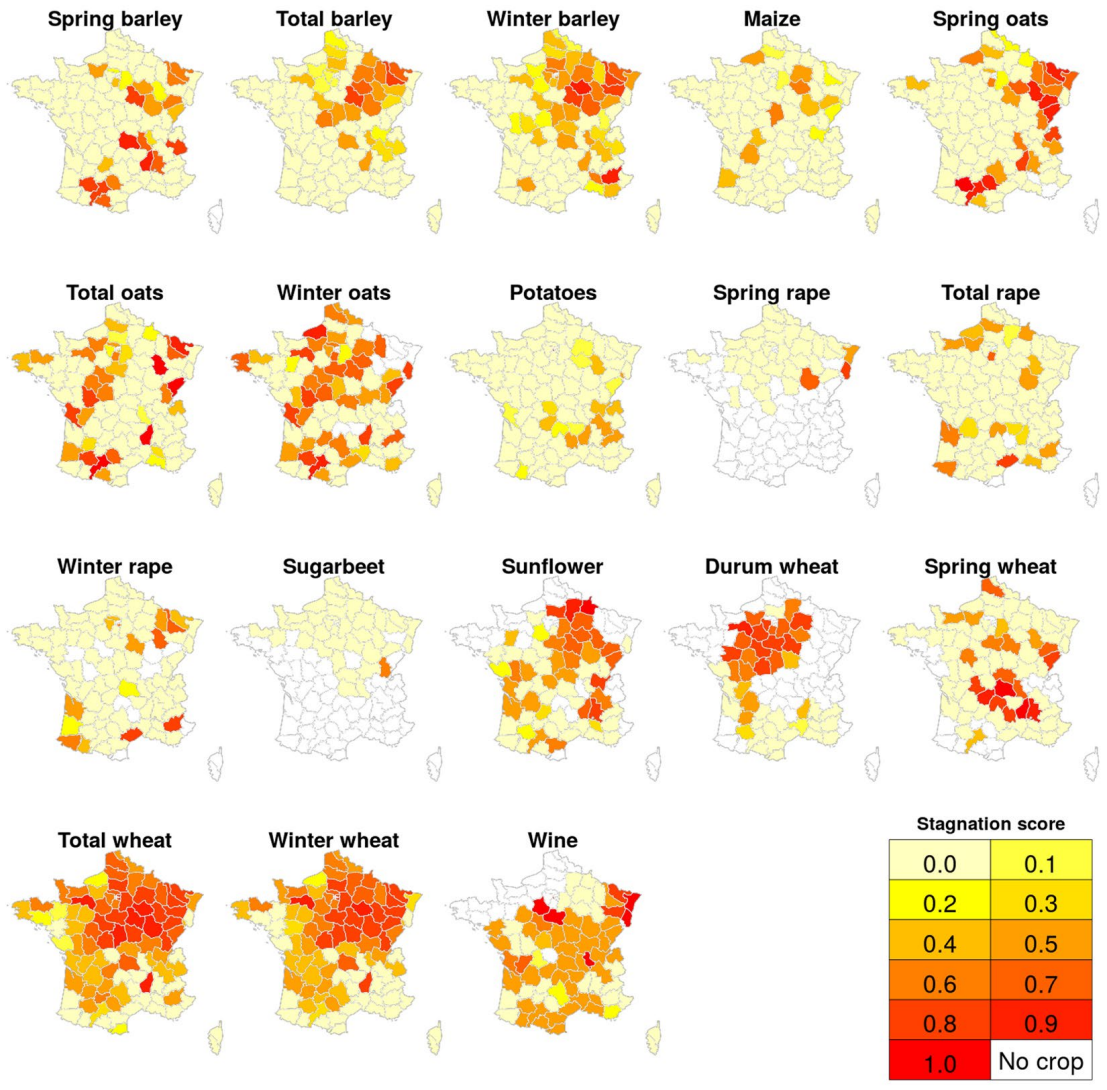
Likelihood of yield stagnation per crop and department in recent years (1997–2016), expressed as stagnation score. Shades of yellow to red correspond to increasing scores, indicated in the legend. A value of 0 indicates “clearly no stagnation” while a value of 1 indicates “clearly stagnating or declining yields”. Blank departments indicate no cropping. See SI Fig. 1 for detailed trend lines of national aggregates and SI Fig. 3 for named departments.

We tested whether we can robustly identify yield stagnation in recent decades. Stagnation is evaluated independently for each crop and department by calculating a stagnation score between 0 (clearly no stagnation) and 1 (clearly stagnating yields), where scores are assigned depending on the value of annual growth rates since 1997, i.e. the last 20 years in our data, relative to a detection threshold of a growth rate of 0.25% per year (see Methods for details). This stagnation score is shown in Fig. 6 and SI Fig. 3.

Stagnation was detected as likely (i.e. the score is at least 0.5; see Methods) for wine in a majority of departments, on 79% of its cropping area summed over departments. For winter wheat, 41 departments experienced a likely stagnation of mean yields, corresponding to 66% of its cropping area in France. For spring wheat, in contrast, only 17 departments (20% of cropping area) display yield stagnation. For total wheat combined, 46 departments or 70% of growing areas are found to have stagnating yields. Likely stagnation of mean yields is also found for all barley types in 13–21 departments (27–39% of areas), all oat types in 21–35 departments (23–43% of cropping areas), durum wheat in 20 departments (26% of area) and sunflower in 24 departments (32% of its cropping area). For all other crops, stagnation occurs in less than 25% of areas, and not at national level. For maize, the cropping area with likely stagnation is only 3%. Winter crops show a likely stagnation more often than their spring counterparts. Area shares with likely stagnation are limitedly sensitive to detection threshold or detection start year (SI Fig. 5).

The year where mean yield growth turned to stagnation is very consistent across crop species and lies, on average, around 1998 (range is 1997–2004) for barley, 1998 (1997–2004) for oats, 1997 (1997–2000) for sunflower, 1998 (1997–2003) for soft winter wheat, 1999 (1997–2002) for durum wheat, and 1997 (1997–1997) for wine. A map with the onset years of stagnation is provided in SI Fig. 4. Note that testing only started in 1997 here.

For most crops, stagnation occurs preferentially in departments with high average yields. Average productivity levels in departments that show yield stagnation are significantly (*p* < 0.05) higher than those in departments that show no stagnation: for winter barley mean yield in departments with stagnation is 6.28 t/ha (+/−standard deviation of 1.22 t/ha) while in those without it is 5.58 t/ha (+/−1.28 t/ha). This pattern is equal for winter oats (stagnating: 4.55+/−1.04 t/ha vs. non-stagnating 4.07+/−1.13 t/ha), sunflower (stagnating: 2.47+/−0.28 t/ha vs. non-stagnating 2.29+/−0.33 t/ha), soft winter wheat (stagnating: 7.02+/−1.06 t/ha vs. non-stagnating 5.43+/−1.37 t/ha) and durum wheat (stagnating: 5.81+/−0.49 t/ha vs. non-stagnating 4.4+/−0.96 t/ha). For wine, there is no significant difference between departmental yields with or without stagnation.

## Discussion

Mean yields have grown rapidly over the second half of the 20^th^ century and are now several times larger than yield levels around 1900. Minimum and maximum yields increased, too, indicating that yield losses have been better mitigated (higher minima) and that yield potentials did increase (higher maxima). But these trends have stalled in recent decades on a substantial fraction of the cultivated area (at least one quarter) for several crops: wine, winter wheat, barley, oats, durum wheat and sunflower. This is, in particular, not the case for maize. A stalling in growth rates is also observed for maximum yields in the majority of those crops. Minimum yields are not increasing further in some of the stagnating crops, possibly indicating that harvest losses are already at a basal level that is hard to decrease further.

The very high correlation between trends of yields and of N and K_2_O fertilizer application is to be expected given the strong negative influence of insufficient nutrient supply on average yield levels^15^ (though fertilization timing and crop-specific dosage are not recorded in this departmental data set). Durum wheat and wine show lower correlations with N, and durum wheat even negative correlation with K_2_O trends. This may indicate that lacking nutrients are not the main yield-limiting factors for wine and durum wheat. Yield trends are not correlated with P_2_O_5_ supply, though, and again even strongly negative for durum wheat. A possible explanation is the shape of the trend for P_2_O_5_ application (SI Fig. 11): it decreases substantially after 1980, but yields do not – indicating that the comparably low amount of P_2_O_5_ addition is already sufficient for current yield levels, as French soils are rich in phosphate^16^, and phosphate is an element that accumulates in soils being adsorbed on clay particles (iron and aluminium oxides) from previous years of application^17–19^. Trend shape may also explain the lower correlation of yields with K_2_O in comparison to N. Since the fertilizer application data used here is not crop-specific, it is difficult to draw conclusions about nutrient supply and yield trends for single crops. A further caveat is that only the application rates of mineral fertilizer were considered, but organic fertilizer application can be equally important in some departments^16^. Finally, increased fertilizer application rates are not the only reason for increased yields, as enhanced genotypes, changing soil attributes and management choices also play a decisive role.

The Northern parts of France seem to offer consistently better growing conditions for crops, evidenced by significantly higher mean yields, with maize and wine as the only exceptions. Reasons for better crop growth in the North include more favourable temperatures (especially for winter crops), higher precipitation, deeper soils with high levels of organic matter^20^ and a higher fertilizer use (SI Fig. 10). Given the uneven distribution of crop performance in France, it would be interesting to evaluate whether the current allocation of crops and croplands is optimal – similar to a study by Ben-Ari and Makowski^21^ who calculated stability gains in major crops by re-distributing crop land fractions over the globe into regions with lower variability. The widening of the intra-country productivity gap (between clusters) over time may indicate that non-climatic and non-edaphic growing conditions could be improved in regions showing low yield levels. Furthermore, follow-up investigations on, for example, the share of weather-dependent yield variation, may benefit from stratifying the country into productivity regions to avoid blending of results with confounding non-weather factors.

The increase in absolute yield variability (standard deviation), but decrease in relative variability (CV) over the last century is presumably driven by the increase of genetic potential and thus maximum attainable yields, and by the prevention of severe loss events, likely due to more intensive management (irrigation, fertilizer use, fungicide and herbicide applications). For barley, oats, sunflower and wine, however, recent decades have shown no further decrease of relative yield variability, and even an increase in some departments – which is consistent with slower growth or stagnation of yields. Increased yield variability in recent years (in contrast to an overall decrease since 1900) may be due to increased climate variability, which has to be investigated in a further study. Absolute yield variation may be related to irrigation, as claimed by Hawkins, *et al*.^11^, but the results obtained here are counterintuitive (SI Fig. 13). Though some crop standard deviations are significantly correlated with the nationally aggregated area equipped for irrigation (from FAO^4^), the correlation direction is positive and thus unrealistic as more irrigation is expected to reduce yield variation^11^. The two crops with negative correlation are sunflower and wine, which are rarely irrigated in France. Furthermore, maize standard deviation is increasing over time, and not diminishing as stated by Hawkins, *et al*.^11^. Overall this analysis highlights the importance of sub-national analyses of crop growth with crop-specific irrigation data (which are not yet available) rather than on aggregate national level.

Yield stagnation is detected on substantial fractions of the cropping area for major crops, and is more likely in departments with high average yields. Stagnation could be caused by several factors. First, a physiological yield potential could have been reached^22^. If stagnation is due to asymptotically decreasing growth when reaching the genetic potential, this would manifest in three further observations: (i) a stalled growth for maximum yields, (ii) higher inter-annual relative variation (i.e. higher CV due to the negative skewness of yield residuals; although higher yield variability may also be caused by more variation in climate, we assume stagnation as an additional, independent source of increased yield variation) and (iii) higher average yields in departments with stagnation as compared to those without stagnation. Except for winter wheat (see also below), none of the crops detected as stagnating fulfils all three criteria.

Second, climatic conditions could recently have changed such that no further increase of crop yields is possible without adequate adaptation, even if the genetic potential was not yet reached. An increased sensitivity of crops to climate variation could be an indicator for a climatic cause of stagnation. The still increasing minimum yields, though, suggest that losses due to adverse climate are not a major reason for mean stagnation (except for wine and sunflower). Previous assessments have indicated that climate change is already visible in crop trends^11,12,23–25^ – thus the question of how much climate changes causes crop stagnation should be further studied.

Third, political decisions, for example in the Common Agricultural Policy (CAP) of the European Union, and an ensuing change of financial incentives or quotas for certain crops could have contributed to lower investment in breeding or a decrease in input use. An example for arbitrary limitation of yield growth is wine: wine yields are stagnating in many French departments. But (reported) yields are kept at an upper threshold for two reasons: limitations of the regional wine-growers labelling associations (termed *AOC* for *Appellation d’Origine Contrôlée*) that install a cap on the amount produced, and a preference of the market for quality rather than quantity. An analysis of political causes for yield stagnation would therefore be a valuable extension of this study.

Fourth, changes in crop rotation (like a lower share of legumes or increasing shares of serial monoculture) or soil carbon content could have contributed to stalled growth, as already speculated by Brisson, *et al*.^5^. For these assessments, detailed and time-resolved data sets on crop rotation and soil carbon contents are required, which are currently not available on the required spatial level.

Fifth, marginal costs for management interventions could have reached a balance where a further investment into crop production, for example with fertilisation or irrigation, does not pay off at harvest time – resulting in a stable level of both management input and yields, but no further harvest increases. This may partly be the case for potatoes and rapeseed, where yields in departments with stagnation are lower (significantly only for potatoes), which may hint to a secondary importance of these crops and also less investment therein. Changing demand for certain crops may also account for stalling investments and ensuing yield stagnation. The necessary data set on management input for such an analysis does, to our knowledge, not yet exist. The fertilizer data used here is not crop-specific and does not permit such a detailed analysis of reduced input leading to reduced increases.

Sixth, and finally, an increase of relative area share in favour of organically grown crops may lead to average yield stagnation as organic yields are usually lower than those on conventional fields^26,27^. Yet the share of organic agriculture in France is only about 2.8% for cereals^28^, such that an increased share of organic agriculture is not assumed as a major reason for stagnation.

Previous studies on yield stagnation in France only treated wheat and maize. The geographic pattern of wheat stagnation detected by Michel and Makowski^9^ is comparable to ours (Fig. 4). Brisson, *et al*.^5^, though analysing only a subset of departments, find stagnating wheat yields for most areas in their subset, and especially in regions with higher average yields – which is in line with our findings. On the national level, we detect a stagnation of wheat yields (SI Fig. 3), in accordance with Calderini and Slafer^6^, Lin and Huybers^8^ and Grassini, *et al*.^10^. Ray, *et al*.^7^ stated wheat yields as stagnating in 80% of crop areas – which is slightly higher than our estimate of 70% of its cropping area (spring and winter wheat combined). Regarding maize, Ray, *et al*.^7^ state to find no detectable stagnation in 90% of the area, where we similarly find 97% as non-stagnating, while Grassini, *et al*.^10^ identified maize as stagnating on national level – which we do not (despite national yields increasing at lower rates or occasionally decreasing after 2000; Fig. 1). The selection of the best-fitting model among different linear formulations, as practiced by Grassini, *et al*.^10^, may thus not be apt for such analysis due to too rigid model formulations.

For sunflower, French growers assume a multitude of possible causes for stagnation, among which are slow genetic potential increase, climate change, non-optimal management on low-yielding soils or monoculture tendencies. It seems, though, that genetic progress for sunflower in experiments has been faster than on actual fields^29^, such that further explanations are necessary.

In sum, there has been consensus on wheat yield stagnation in a majority of French cropping areas, and we add further evidence to this. For maize, we find a stagnation in only few cropping areas and thus refute the statement by Grassini, *et al*.^10^ that maize yields have not improved in recent decades. Our results additionally show that stagnation is not limited to winter wheat but also affects substantial area shares of other crops.

The hypothesis of reaching a yield potential can only serve as a putative, but uncertain explanation for stagnating yields in the case of winter wheat, as there are hints from field trials that genetic gain in wheat yield potential has not stalled^5,9^. For all other crops that show stagnation further research for the cause(s) is needed, currently impeded by lacking data. Given this lack of knowledge, an outlook on future yield growth rates is furnished with uncertainty. It is obvious that an assumption of sustained yield growth into the future would at best be naïve, even when not accounting for climate change as an additional danger for harvests.

Our stagnation detection method, using a flexible scoring scheme, allows for assessing uncertainty of stagnation and is comparable between crops and departments. The stagnation score depends on the chosen absolute threshold of growth rates, and on the width of the temporal window used for testing (SI Fig. 5). But our results are only altered quantitatively, not qualitatively – that is, a substantial fraction of cultivation areas suffers from non-growing yields under any definition of stagnation. The choice of 0.25% as a detection threshold clearly distinguishes stagnation from growth rates in times of rapid increase, and also lies substantially below the increase rates deemed necessary for the near future^2,3^. Dependence of results on the choice of stagnation definition and threshold – from which all other studies cited above suffer similarly – is inevitable and reflects the fact that the question of stagnation remains open except for very clear cases.

The split of crops between spring and winter cultivars is of relevance, as in some cases (e.g. barley in Haute Saone) both spring and winter growth is considered as likely stagnating – but not so when yields of both varieties are combined. The reason is a changing area share, from exclusively spring barley (1940’s to 1970’s) to almost entirely winter barley in recent years. Since winter barley yields are, on average, higher than those of spring barley, the combined harvest of both crops keeps increasing when spring barley is replaced by winter barley, even when winter barley itself is not increasing any more. A further promotion of winter cultivars, where weather is appropriate, may therefore be helpful for sustaining combined yield growth. Such a split between spring and winter varieties is not usually performed in other studies, despite its obvious practical relevance.

During the time frame of this data set, the two world wars (1914–18 and 1939–45) occurred, with significant aftermaths for economic and agricultural life. The turmoil of these times is reflected in the data, with lower absolute yields (Fig. 1) and higher variability (Fig. 4), but also strong growth after the war in the 1940’s and 1950’s (Fig. 2) – possibly due to a base effect of low yields. It is, furthermore, not unlikely that the quality of the census procedure for recording agricultural performance suffered during war times such that values in these years are engrained with higher uncertainty.

In conclusion, French crop yields have developed to higher mean yields and lower relative variability over the full 20^th^ and beginning of the 21^st^ century. The continuation of these positive trends is of importance for local and global food availability, as France is a major producer of several staple crops like maize and wheat, and is exporting to many countries especially in Northern Africa. Yet recent stagnation for some crops, in particular for wheat, calls into question whether positive trends can be maintained, and merits further research into the underlying causes. In performing such examinations, crop-specific fertilizer dosage per department, the outbreaks and severities of pests and diseases (specific for each crop and department), the performance of field trials to assess recent gains in genetic potential, and a correlation with climate data are instrumental.

## Methods

### Data sources and data preparation

Crop yield, area and production statistics from 1900 until 1988 were collected from books of national agricultural statistics (‘Statistique agricole annuelle’ or ‘Annuaire de statistique agricole’) compiled by the French Ministry of Agriculture; detailed references are provided in the supplementary information as an Excel table. Numbers were manually digitized from photocopied versions of the original paper documents. Data from 1989 to 2016 were derived from digital statistics from the Agreste website (agreste.agriculture.gouv.fr; ‘Statistique agricole annuelle’ compiled by the Service de la Statistique et de la Prospective (SSP), Secrétariat Général du Ministère de l’Agriculture, de l’Agroalimentaire et de la Forêt (MAAF), France); details are provided in the supplementary information. Yields were calculated from total production and surface area for each department to avoid apparently often incorrect yield values printed in the old statistics books. Yields are given in kilogram per hectare (kg/ha, for sown area) for dry mass with 10–16% moisture content, depending on the crop.

Data are available for ten crops: soft wheat (French name: *Froment* until 1954*, Blé tendre* from 1955 on; spring and winter separately), durum wheat (*Blé dur*), maize (*Maïs*), oats (*Avoine*; spring and winter), rapeseed (*Colza*; spring and winter), barley (*Orge*: spring and winter), potatoes (*Pommes de terre*), sugar beet (*Betterave*), sunflower (*Tournesol*) and wine (*Vigne*). The split into spring and winter crops eventually results in 18 distinct crop types. Time frames with available data are provided in SI Table 1. Multiple cropping per year within these crops is not usually practiced in France^30^.

The shapes of French departments have changed over time. We use the 96 departments in their current form and subsume historical values to modern departments where possible. Corsica was one single department until 1975 but then split into Corse-du-Sud and Haute-Corse. Yield data for Corsica until 1975 were copied to each of the new departments. Seine and Seine-et-Oise were two departments until 1967, but then subdivided into seven new departments on 1 January 1968. To account for this we consider the values of the seven new departments (Essonne, Hauts-de-Seine, Paris, Seine-Saint-Denis, Val-de-Marne, Val-d’Oise, Yvelines) only from 1968 on and unite the two old departments into one counter-factual until 1967.

### Quality filters

Some yield values had to be considered as outliers, also after checking for digitizing errors. There were three criteria for defining an outlier: unreasonable absolute value (larger than a visually, empirically determined upper bound of 10 t/ha for wheat and barley, 100 t/ha for sugar beet, 15 t/ha for maize, 8 t/ha for oats, 60 t/ha for potatoes, 5 t/ha for rape and sunflower and 200 hl/ha for wine), unreasonable value with respect to total time series mean yield (five times higher or lower than long-term mean, with the exceptions of maize with ten-fold and oats and soft wheat with six-fold outliers), or unreasonable change in comparison with the previous year (more than twenty-fold increase). Outliers were masked as missing values to avoid introducing a bias from any correction. The overall fraction of outliers in the yield data set is 2% (SI Table 1). The fraction of outliers is between 0.1% and 2.8% for all crops except spring barley, spring and winter rape, where they range between 4.6% and 14.5% (SI Table 1).

### Yield data description and quality check

The data set contains 120,602 yield entries after outlier filtering (SI Table 1). For area and production data, the number of entries is 124,280 and 124,527, respectively. The (partly) manual digitization of the almost 370,000 data points was a large effort subject to possible errors. Hence, to evaluate the quality of the data base, we compared national yields (aggregated from department level with area weighting) in our data with FAOSTAT yields^4^ which are available from 1961 to 2016. FAO yields are available for barley, maize, oats, potatoes, rapeseed, sugar beet, sunflower and soft wheat. For crops with distinct spring and winter types only yields averaged over both types were compared to FAO yields. All correlation coefficients between these two datasets were above 0.99 (Pearson’s *r*) and highly significant with p < 1e-5. These high correlations indicate soundness of our data. It has to be considered, though, that FAO statistics are compiled from subnational data in France – thus the two data sets are not independent. The high correlations therefore mainly point to the quality of digitalization. The low fraction of outliers, which we assume to be annotation errors in the statistical yearbooks, further indicates the high standard of the raw data set.

### Trend detection

Trends were detected with Dynamic Linear Models (DLM, Petris, *et al*.^31^), exemplified by Michel and Makowski^9^ to be a robust method for crop trend analysis. DLMs allow an adjustment of trends over time and provide annual growth rates without making strong assumptions about the functional form of yield trends. A DLM is described by an observation equation (relating estimated and observed yields; Equation 1) and a system equation (defining the iterative changes in yields; Equation 2):

1 $${Y}_{t}={a}_{t}+{\varepsilon }_{t}$$

2 $${Z}_{t}=G{Z}_{t-1}+{\tau }_{t-1}$$

where *Y*_*t*_ is observed yield at time *t*, a_*t*_ is smoothed (or estimated) yield level at time *t*, ${\varepsilon }_{t} \sim N(0,\,{\sigma }_{\varepsilon }^{2})$ is a normally distributed error term,

$${Z}_{t}=(\begin{array}{c}{a}_{t}\\ {b}_{t}\end{array}),\,G=(\begin{array}{cc}1 & 1\\ 0 & 1\end{array})\,{\rm{and}}\,{\tau }_{t-1} \sim N(0,\,(\begin{array}{cc}{\sigma }_{a}^{2} & 0\\ 0 & {\sigma }_{b}^{2}\end{array})).$$

Variance parameters ${\sigma }_{\varepsilon }^{2},\,{\sigma }_{a}^{2},\,{\sigma }_{b}^{2}$ were estimated from data by maximum likelihood, then plugged into a Kalman smoother algorithm for estimating *a*_*t*_ and *b*_*t*_. More specifically, variables a_*t*_ (the underlying yield level, defining the yield trend) and b_*t*_ (the annual growth rate) were both defined as time-varying stochastic variables and their values and variances were estimated with a Kalman smoother algorithm^32^. For further details we refer to Michel and Makowski^9^. An independent DLM was estimated for each crop and department, and also for nationally aggregated time series (Fig. 1; SI Fig. 1). National trends were calculated for mean (area-weighted), minimum and maximum and for the 5, 10, 25, 50, 75, 90 and 95% quantiles across departments. Growth rates *b*_*t*_ (estimated with the DLM) were converted from absolute to relative values by dividing them by the estimated yield levels *a*_*t*_. The 90% confidence intervals for growth rates were calculated from the variances provided by the Kalman smoother. Trends and growth rates were only estimated if at least 30 yield observations were available. The R^33^ package ‘*dlm*’ was used for all calculations.

Distributions of yield growth rates over time across all crops and departments were graphically described using a bubble plot (Fig. 2). For each crop and department the mean growth rate per decade was calculated. Afterwards, growth rates were categorized across all crops, departments and decades, with category 1 indicating lowest (negative) growth and category 5 indicating strongest growth. Bubble areas represent the number of crop-department combinations per category, normalized by the total number of values per decade such that the sum of areas over the five categories is equal across all decades.

### Detection of stagnating yields

We tested for recent stagnation of yield growth independently for each crop and each department. Within this study, we define yields as *stagnating* within a given time frame if annual growth rates of yields predominantly stay below a chosen threshold. This also subsumes yield decline, i.e. negative growth, which we do not further distinguish here from stagnation. “Predominantly” implies that not every growth rate needs to stay below the chosen growth rate threshold (SI Fig. 2a). Rather, we applied a scoring scheme based on equivalence tests^34^, using the relative positions of the 90% confidence intervals for annual yield growth rates and an interval of growth rate defined as indicative for stagnating yields. For the latter, we chose an annual yield growth rate of 0.25%, centred in an interval of 0.0 to 0.5% (SI Fig. 2b). For each annual growth rate and its confidence interval, ten distinct positions between growth rate interval and the stagnation interval are possible (SI Fig. 2b). For each of these positions, a score between 0 and 1 was assigned, with 0 indicating growth rates clearly beyond stagnation and 1 indicating clear stagnation. The scores for each year within the testing time frame were summed and divided by the number of years in the test window, resulting in an overall stagnation score. This score ranges from 0 (certainly no stagnation) to 1 (certain stagnation or decline). The testing time frame used in the study comprised the last 20 years of data, 1997–2016. The score can be interpreted as likelihood for yield stagnation. For analysing affected national areas, a yield time series in one department was defined as “likely stagnating” if the overall score was at least 0.5 (i.e. 50% or more of the years in the time frame indicated stagnation). In the case of stagnation, the onset year was defined as the first year where yield growth rate was lower than the upper range of the stagnation test window (i.e. 0.5% per year). Sensitivity of the results with respect to detection threshold and time frame was performed, with winter wheat as emblematic crop (SI Fig. 5).

### Spatial clustering of departments

Spatial coherence between departments with respect to mean yields was studied by clustering departments in four consecutive time frames (1900–1929, 1930–1959, 1960–1989, 1990–2016), using the distance between mean yields (averaged over the respective time frames) as measure of dissimilarity. A hierarchical clustering with UPGMA (Unweighted Pair Group Method with Arithmetic mean) was used, grouping those departments with minimum unweighted Euclidean distance in mean yields. Leftmost and rightmost 1% of data were removed before clustering to avoid a domination of outliers. The optimal number of clusters was determined separately for each crop and time frame, using the Calinski-Harabasz measure (CH-Index) of cluster quality, which maximizes inter-cluster and minimizes within-cluster variance^35^.

### Correlation of yield trends with fertilizer trends

Yield time series were correlated with department-specific, but not crop-specific, mineral fertilizer usage (1946 to 2013). Total application of mineral N, P_2_O_5_ and K_2_O (in tonnes) was available, also digitized from the *Statistique Agricole Annuelle* (as for yields) from 1946 to 1988, and from the UNIFA (*Union des Industries de la Fertilisation*) from 1989 to 2013. The Pearson correlation coefficient between yields and fertilizer doses was estimated for each crop and department, once with the absolute yield values and once with de-trended yields and fertilizer time series.

### Analysis of yield residual variability

Inter-annual yield variability was studied using the yield residuals, i.e. the values that remain after subtracting the trend (estimated from DLM) from the original values. To assess the temporal evolution of yield inter-annual variability, the decadal Coefficient of Variation (CV) was used, defined as the standard deviation (of the residuals) over the mean (of the original yield data). Similarly as for growth rates, a bubble plot was used to visualize the evolution of CV over decades, thereby merging all crop-department cases (maximum of 18 * 96 possible values) per decade.

The Pearson correlation coefficient of yield residuals between crops was estimated from national yield mean and percentiles (5% and 95%) of residuals for all pairs of crop species, over the common time frame with available data for each pair of crops.

### Sensitivity to removal of departments with small cropping area

All analyses described above were first performed with the full data set and then with a restricted data set where departments with a low crop-specific cropping area (mean area below the 10% quantile of mean area across all departments, for a specific crop) were removed to test the sensitivity of our results. As a summary outcome of this sensitivity test, results are very similar to those without omission, and none of the conclusions stated in the manuscript changes when the 10% departments with smallest cropping area are removed. Results are not shown due to space constraints.

## Electronic supplementary material

Supplementary Information

Dataset 1

## Data Availability

The data on French agriculture are publicly available at http://agreste.agriculture.gouv.fr from 1989 on. Data before 1989 are available from the corresponding author on reasonable request.
