# Supplementary material for: Yield trends, variability and stagnation analysis of major crops in France over more than a century
Source: Sci Rep. 2018 Nov 15;8:16865. doi: 10.1038/s41598-018-35351-1 (PMC6237926; doi:10.1038/s41598-018-35351-1)
Supplement: Supplementary file 1 — Supplementary Information [file 41598_2018_35351_MOESM1_ESM.pdf]

Supplementary Information to

“Yield trends, variability and stagnation analysis of major crops in France over more than a century”

By B. Schauberger, T. Ben-Ari, D. Makowski, T. Kato, H. Kato, P. Ciais

Supplementary Figures

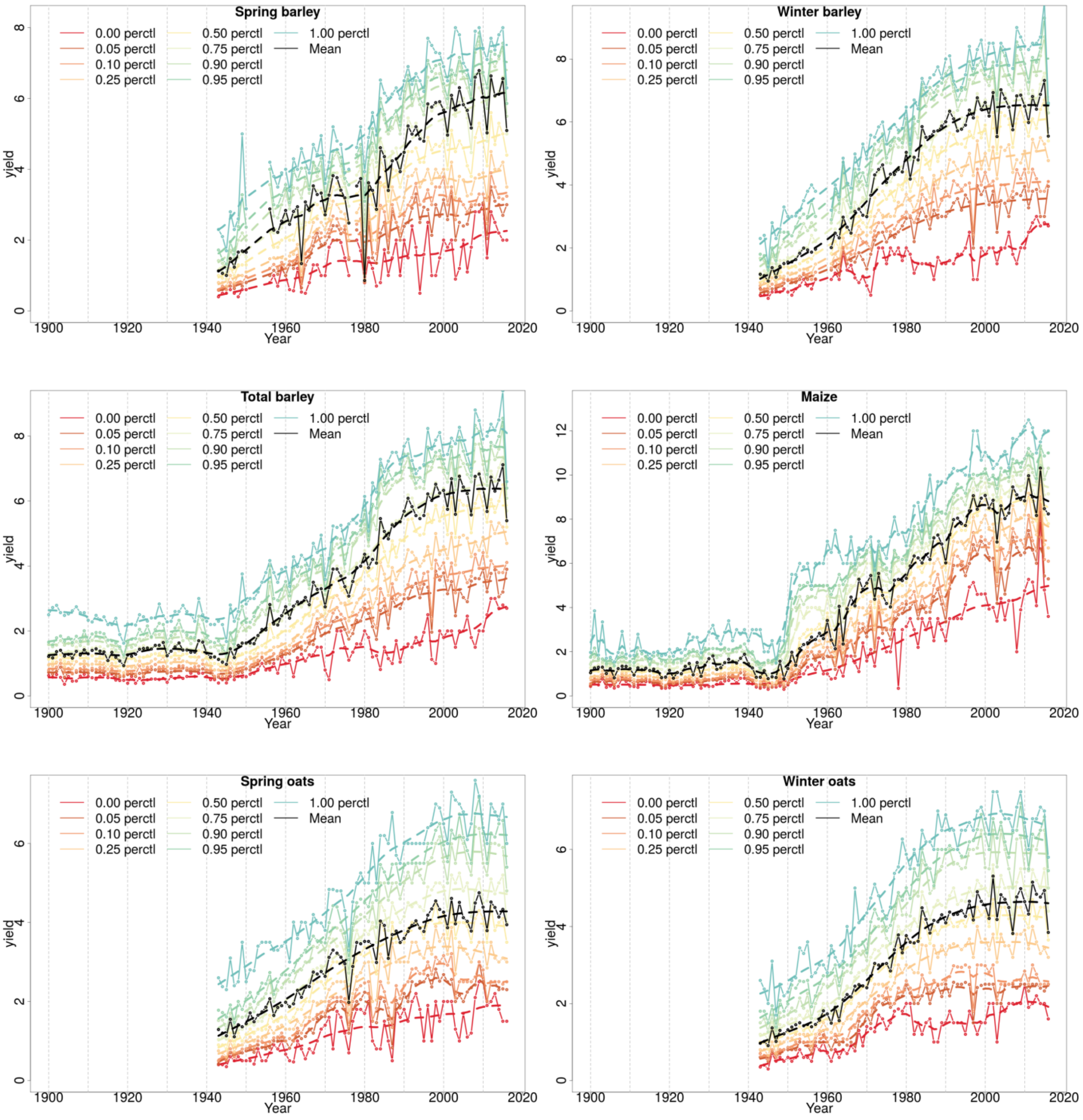

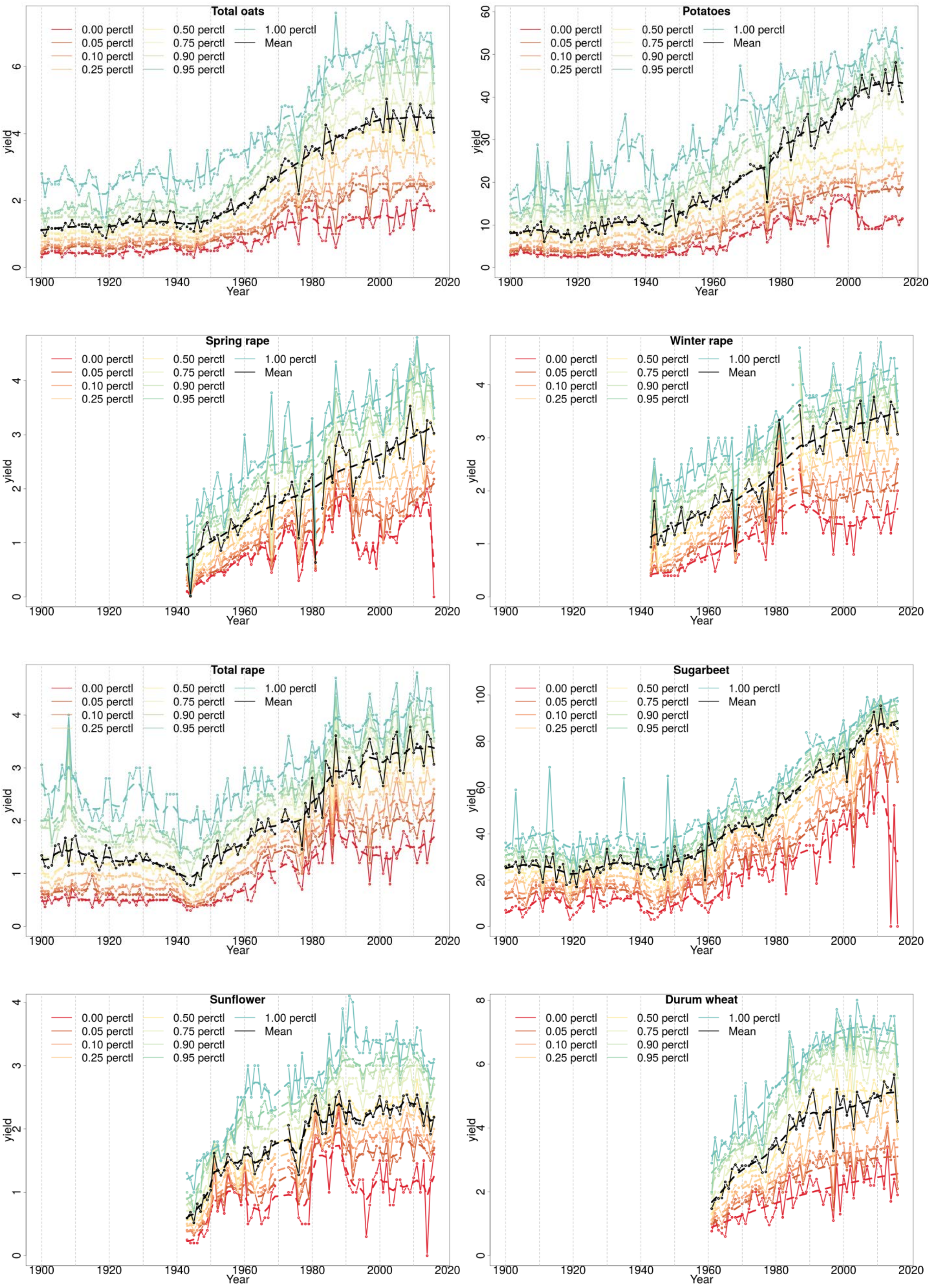
Total oats
Potatoes
Spring rape
Winter rape
Total rape
Sugarbeet
Sunflower
Durum wheat
0.00 perctl
0.05 perctl
0.10 perctl
0.25 perctl
0.50 perctl
0.75 perctl
0.90 perctl
0.95 perctl
1.00 perctl
Mean
yield
Year

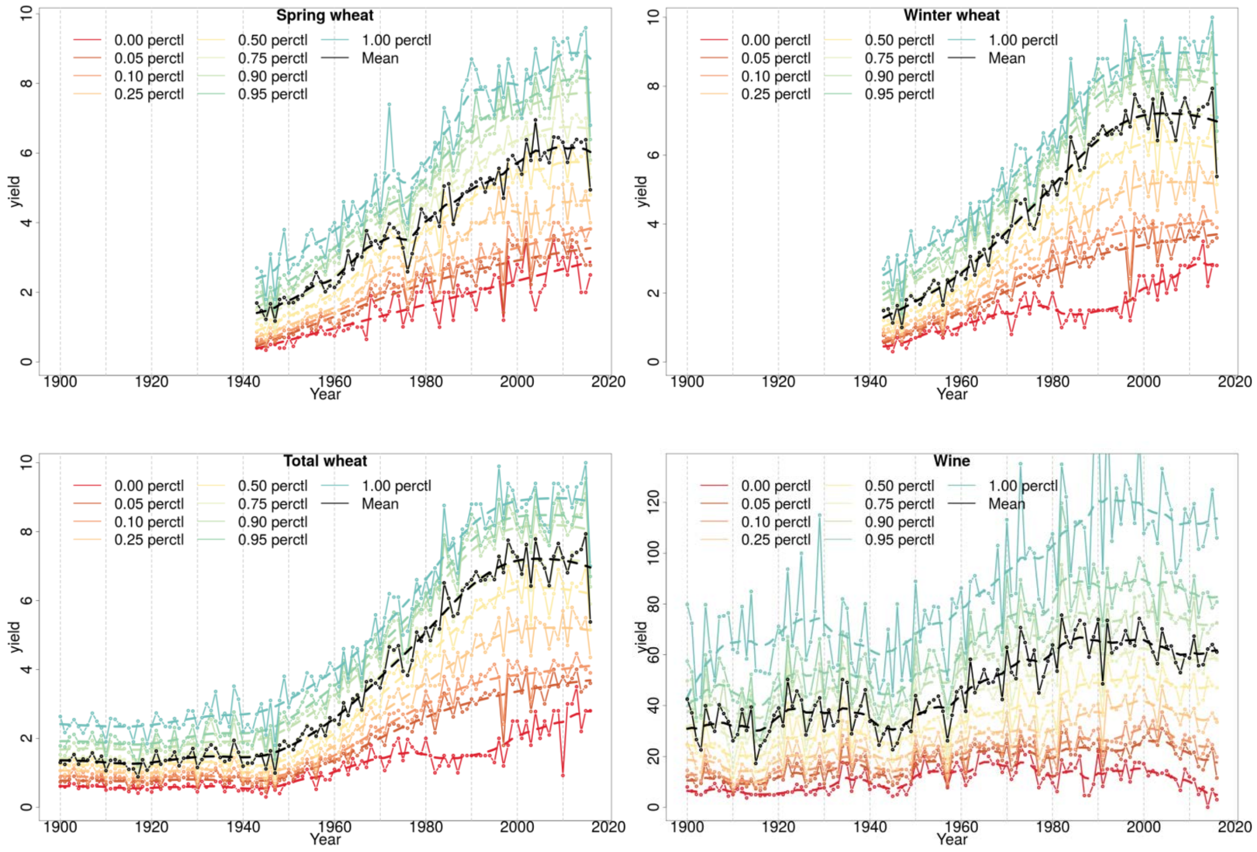

SI Figure 1: National yield percentiles across departments for each year, for all 18 crop types.

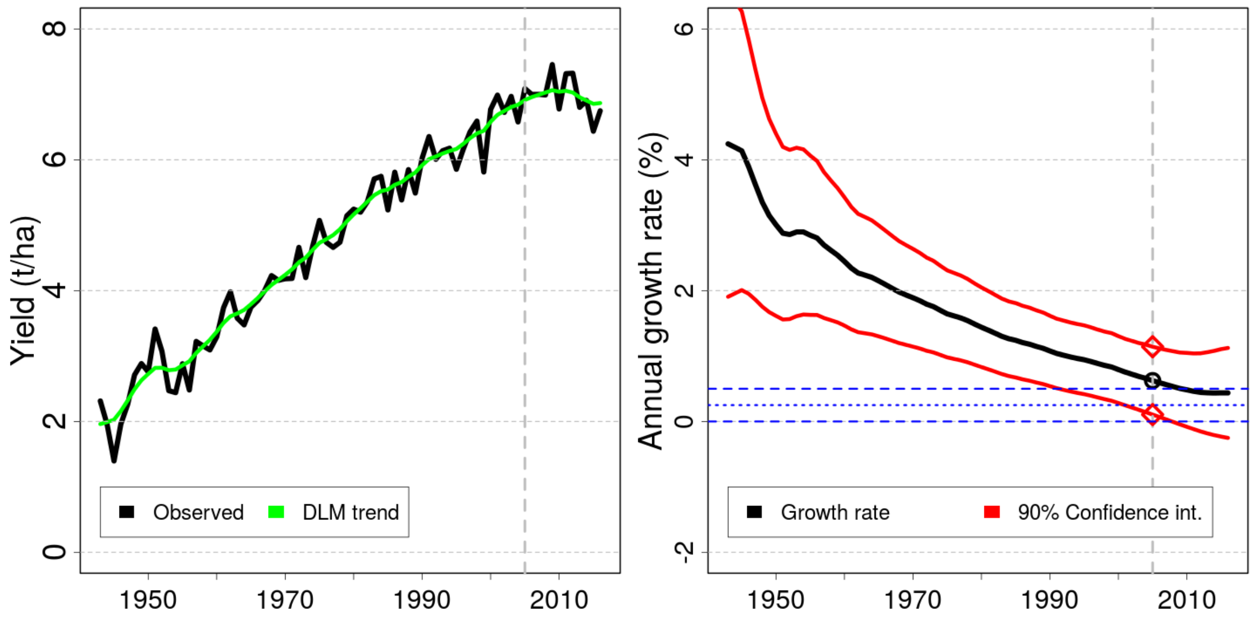

(a)

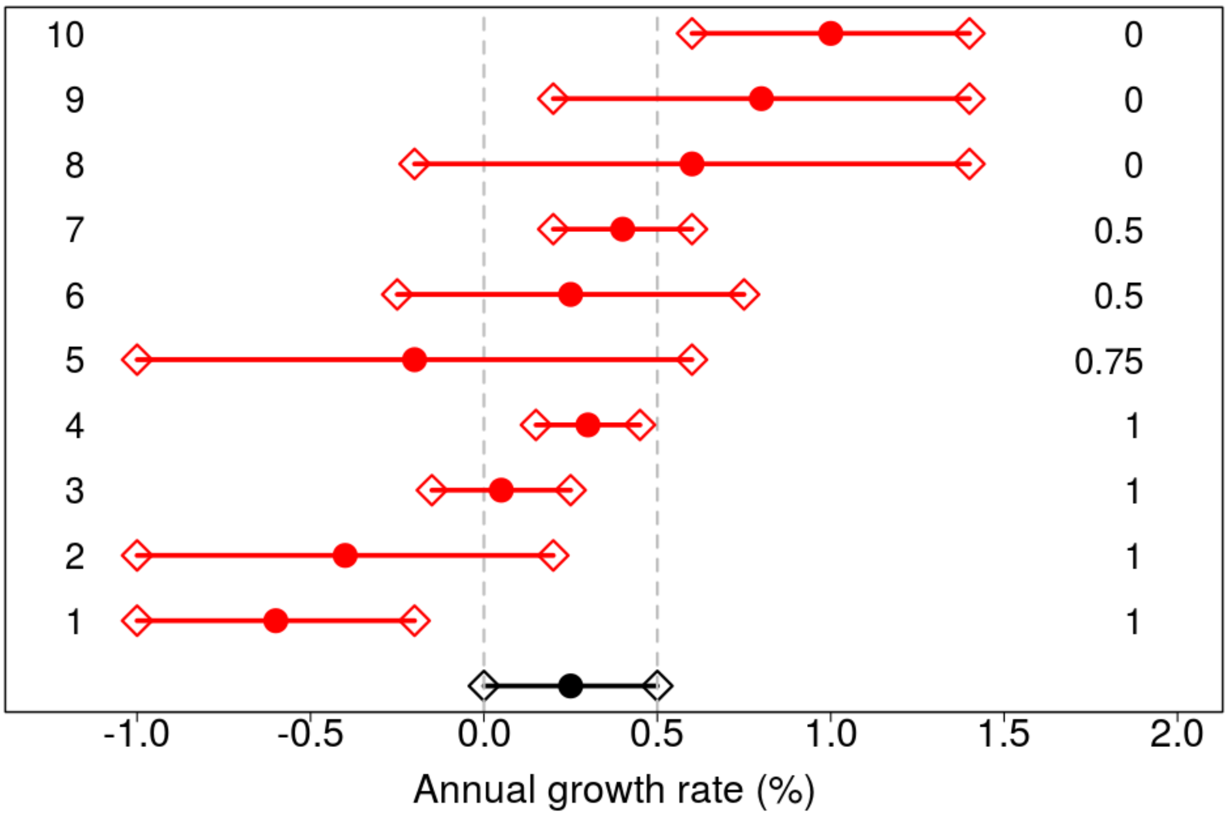

(b)

SI Figure 2: Illustration of the scoring scheme for detecting yield stagnation. (a) Example time series with absolute yields (left panel; black lines = yields, green line = DLM-estimated trend) and growth rates with 90% confidence intervals (right panel; black lines = estimated growth rates, red lines = CIs). Blue dashed lines mark the stagnation detection threshold range while

the grey vertical line marks an example year. (b) Score assignment for the ten different positions of annual growth rates. An interval is defined for growth rates, representing a stagnation threshold by treating observed growth rates within or below as stagnating (subsuming also declining; black lines). Here, we use 0.25% (+/- 0.25) as stagnation threshold and interval. For a time series of annual yield growth rates, the relative position of each year's growth rate with respect to the threshold interval results in a score for this year. There are ten different relative positions given a point estimate (red dots) and 90% confidence intervals for growth rates (red lines with diamonds). Each of them is associated with a score between 0 and 1, which is higher for a higher chance that the growth rate is truly indicating stagnating yields. Finally, annual scores are summed and divided by the number of years in the testing window, resulting in an overall score between 0 and 1 that can be interpreted as a likelihood of yield stagnation. The example year in panel (a) falls into case 9 and is therefore assigned a score of 0.

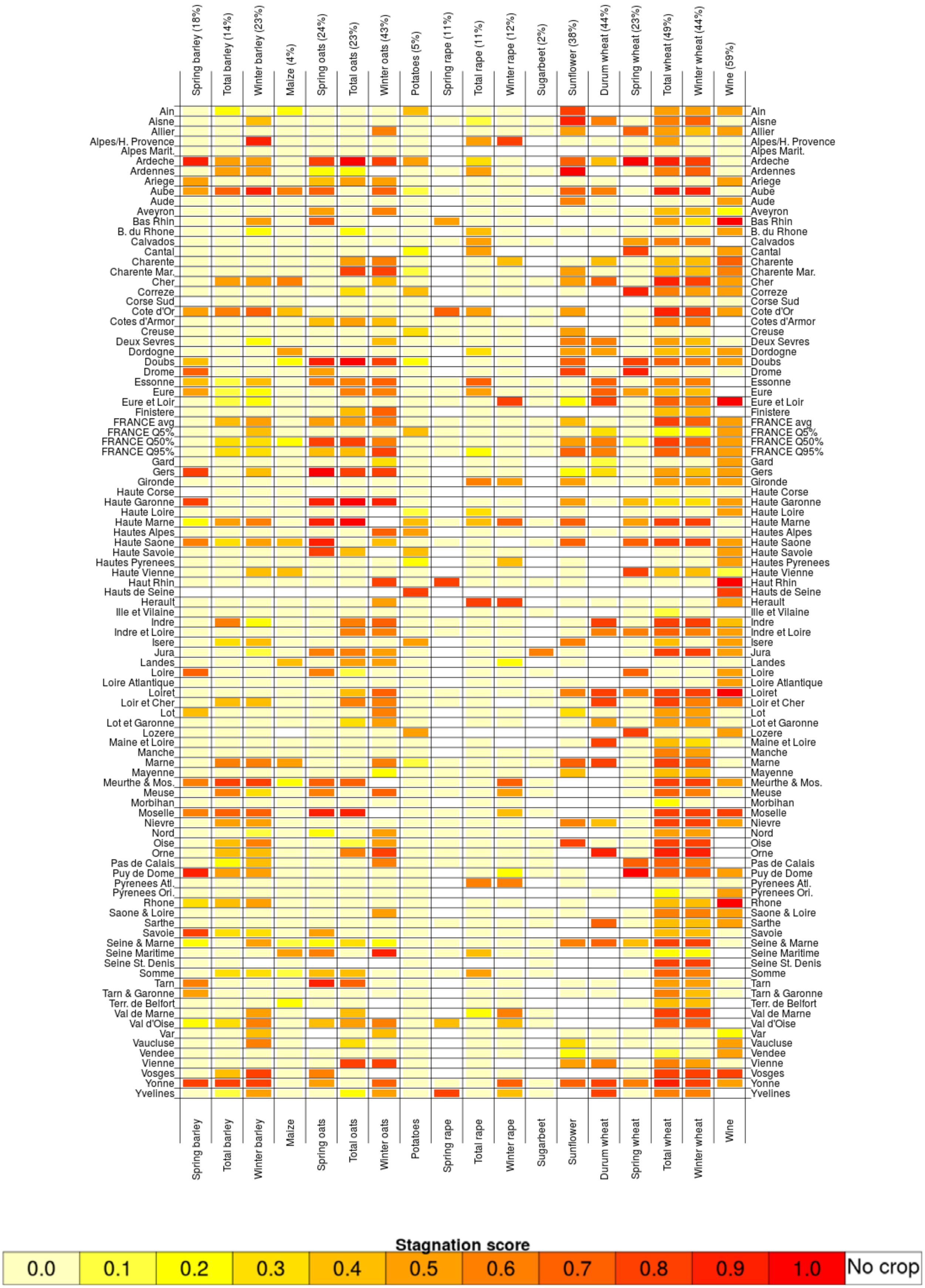
Spring barley (18%)
Total barley (14%)
Winter barley (23%)
Maize (4%)
Spring oats (24%)
Total oats (23%)
Winter oats (43%)
Potatoes (5%)
Spring rape (11%)
Total rape (11%)
Winter rape (12%)
Sugarbeet (2%)
Sunflower (38%)
Durum wheat (44%)
Spring wheat (23%)
Total wheat (49%)
Winter wheat (44%)
Wine (59%)
Ain
Aisne
Allier
Alpes/H. Provence
Alpes Marit.
Ardeche
Ardennes
Ariege
Aube
Aude
Aveyron
Bas Rhin
B. du Rhone
Calvados
Cantal
Charente
Charente Mar.
Cher
Correze
Corse Sud
Cote d'Or
Cotes d'Armor
Creuse
Deux Sevres
Dordogne
Doubs
Drome
Essonne
Eure
Eure et Loir
Finistere
FRANCE avg
FRANCE Q5%
FRANCE Q50%
FRANCE Q95%
Gard
Gers
Gironde
Haute Corse
Haute Garonne
Haute Loire
Haute Marne
Hautes Alpes
Haute Saone
Haute Savoie
Hautes Pyrenees
Haute Vienne
Haut Rhin
Hauts de Seine
Herault
Ille et Vilaine
Indre
Indre et Loire
Isere
Jura
Landes
Loire
Loire Atlantique
Loiret
Loir et Cher
Lot
Lot et Garonne
Lozere
Maine et Loire
Manche
Marne
Mayenne
Meurthe & Mos.
Meuse
Morbihan
Moselle
Nievre
Nord
Oise
Orne
Pas de Calais
Puy de Dome
Pyrenees Atl.
Pyrenees Ori.
Rhone
Saone & Loire
Sarthe
Savoie
Seine & Marne
Seine Maritime
Seine St. Denis
Somme
Tarn
Tarn & Garonne
Terr. de Belfort
Val de Marne
Val d'Oise
Var
Vaucluse
Vendee
Vienne
Vosges
Yonne
Yvelines
Spring barley
Total barley
Winter barley
Maize
Spring oats
Total oats
Winter oats
Potatoes
Spring rape
Total rape
Winter rape
Sugarbeet
Sunflower
Durum wheat
Spring wheat
Total wheat
Winter wheat
Wine
Stagnation score
0.0
0.1
0.2
0.3
0.4
0.5
0.6
0.7
0.8
0.9
1.0
No crop

SI Figure 3: Likelihood of yield stagnation in recent decades (1997-2016), expressed by the stagnation score. Correspondence between colors and certainty of stagnation is given in the legend below. White indicates no cultivation area for the respective crop. National aggregates are displayed as "FRANCE …". The percentage behind crop names represents the share of departments that show stagnating yields, i.e. the score is at least 50%. An excellent map of the names of departments can be found on Wikipedia: https://en.wikipedia.org/wiki/Departments_of_France

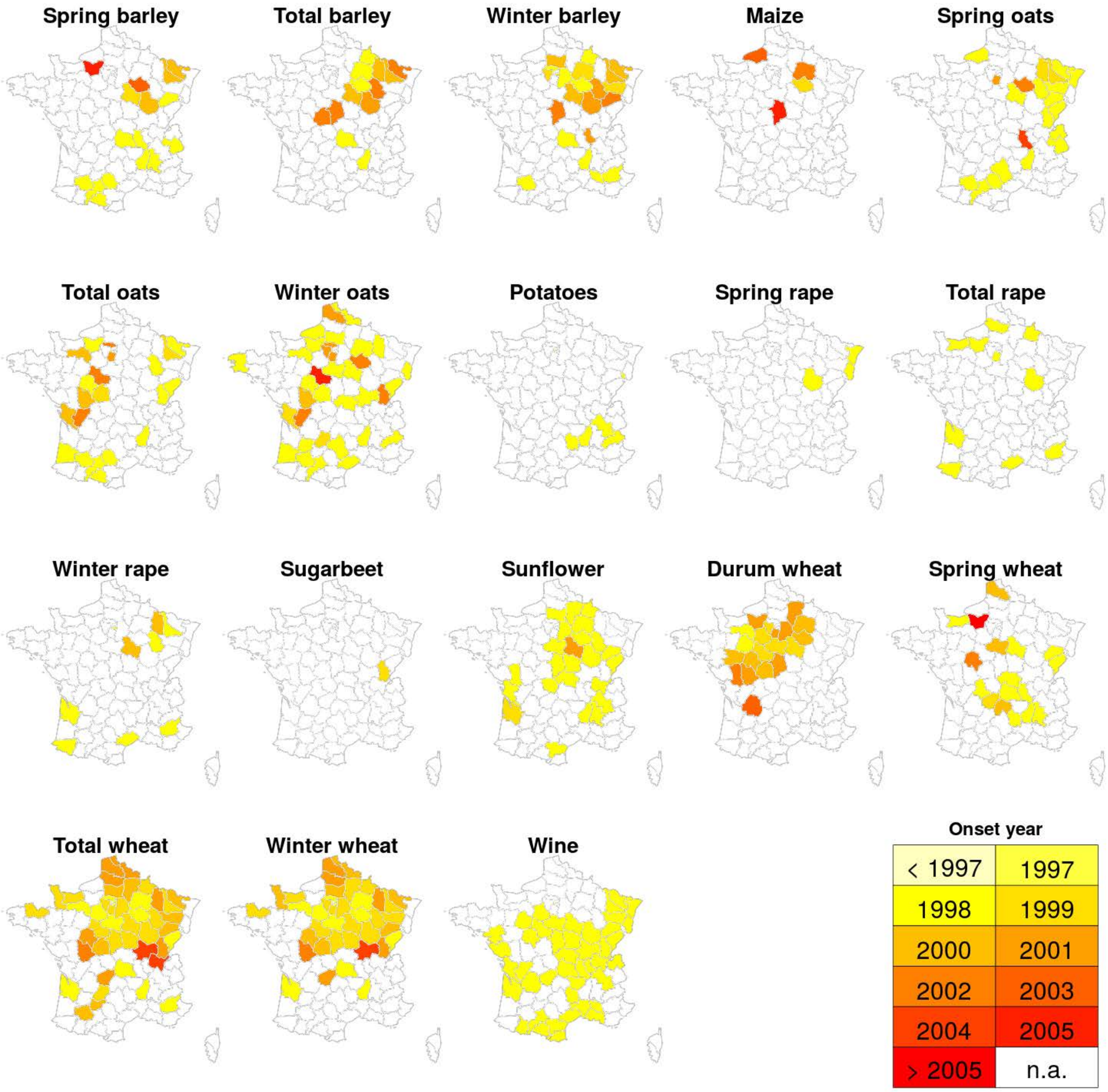

SI Figure 4: Onset year of stagnation. Only departments where yields where robustly detected as stagnating are colored; others are left blank (n.a. = not available; indicating either no cropping or no stagnation).

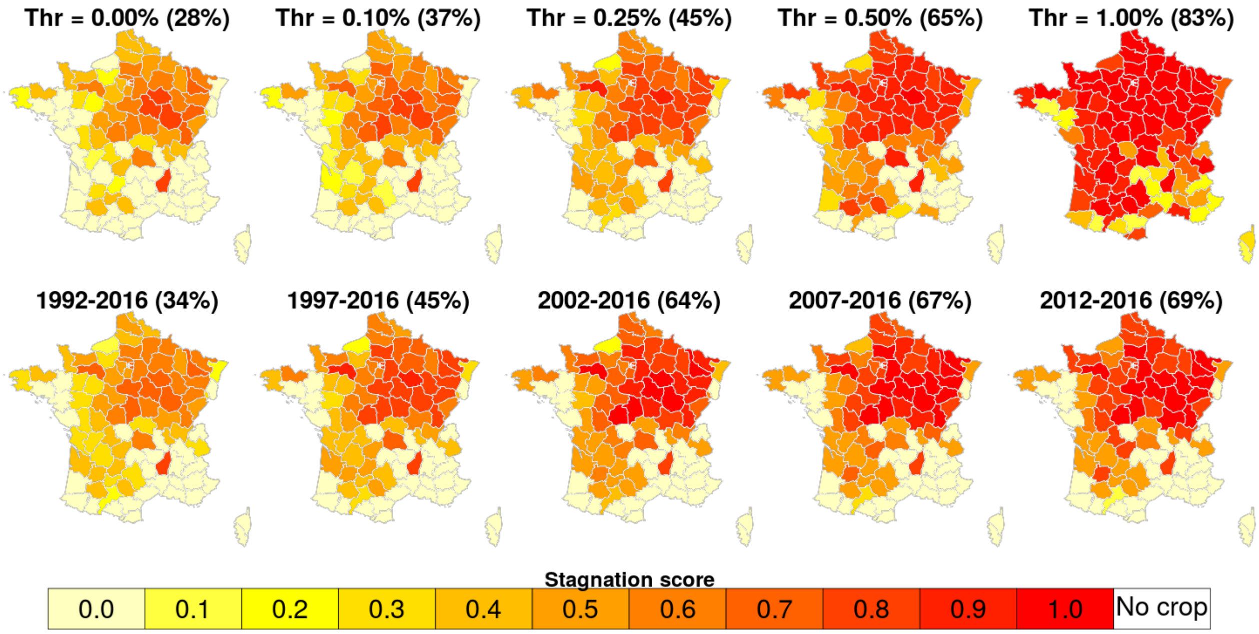

SI Figure 5: Sensitivity of the likelihood of winter wheat stagnation with respect to detection threshold (first row) or time frame (second row). Detection thresholds are varied between growth rates of 0% to 1%, always with +/- 0.25; the time frame in the first row is 1997-2016. Time frames were varied between the last 25 to 5 years; the detection threshold in the second row is 0.25% (+/- 0.25). That means the third image in the first row and the second image in the second row are equal, and also equal to the winter wheat image in main Figure 6. The likelihood color code is given in the legend below. The percentages behind the panel titles represent the share of departments (with winter wheat cultivation) where stagnation is detected, i.e. the score reaches at least 0.5.

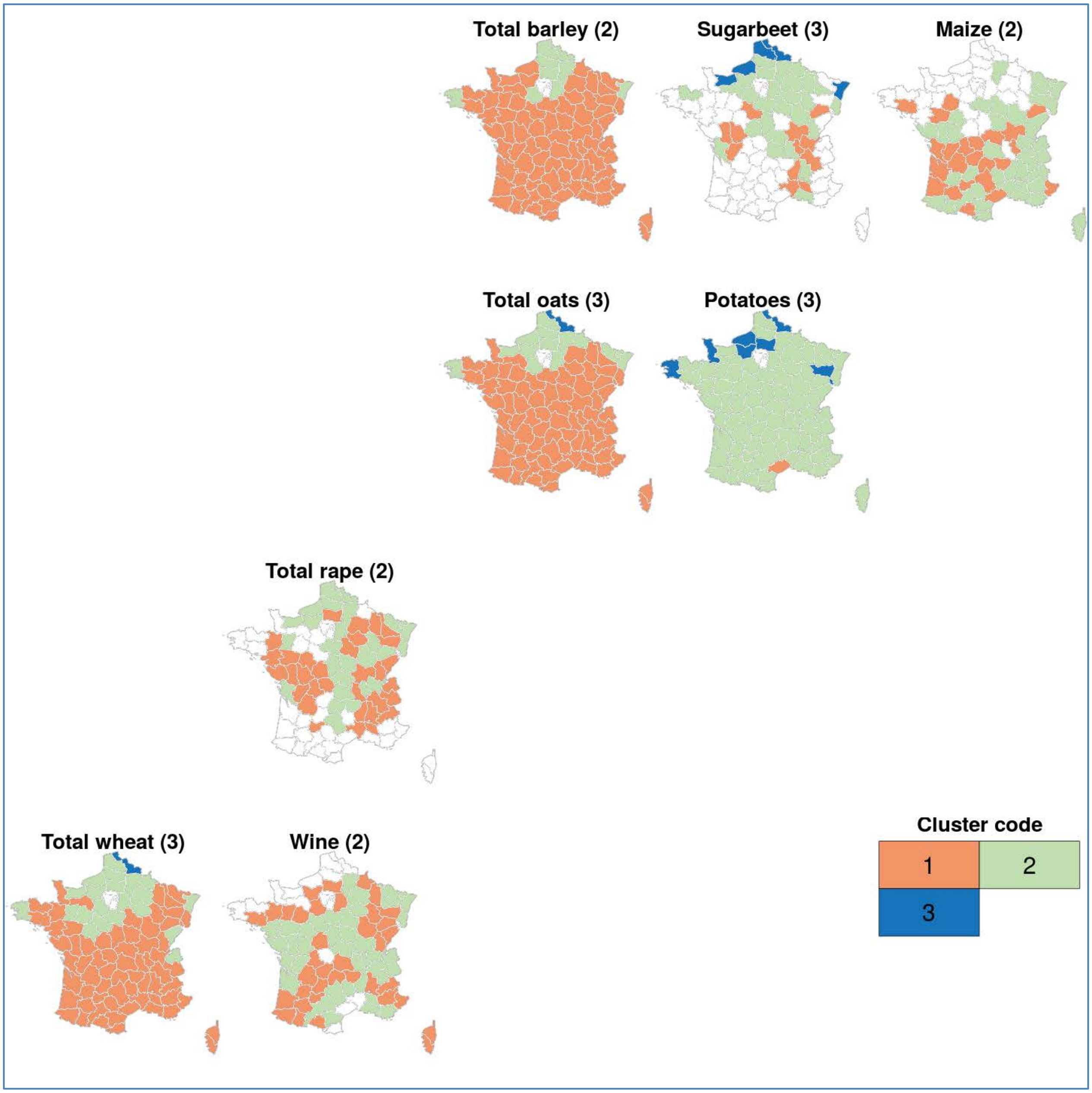
Total barley (2)
Sugarbeet (3)
Maize (2)
Total oats (3)
Potatoes (3)
Total rape (2)
Total wheat (3)
Wine (2)
Cluster code
1
2
3

(a) 1900-1929

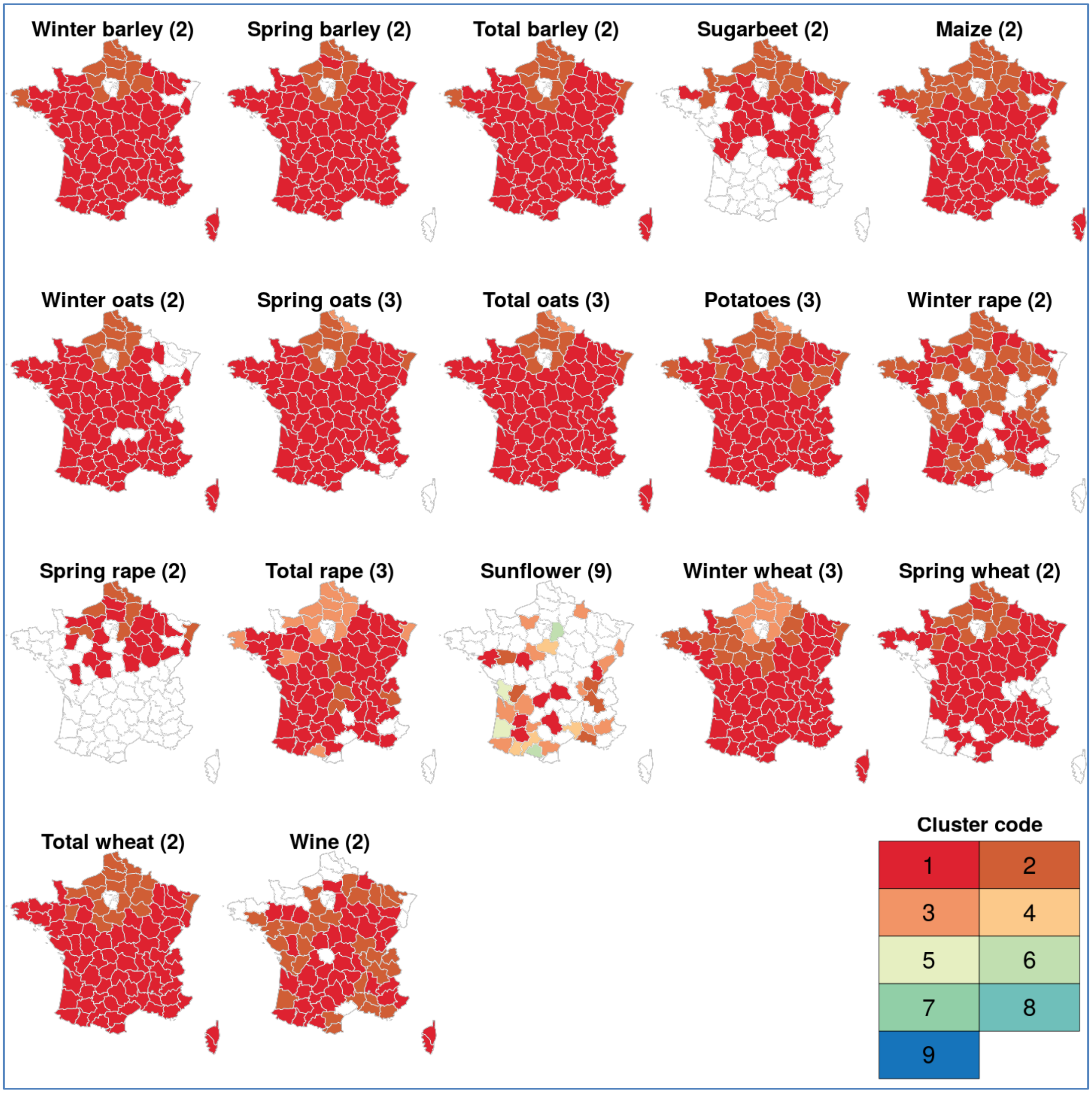
Winter barley (2)
Spring barley (2)
Total barley (2)
Sugarbeet (2)
Maize (2)
Winter oats (2)
Spring oats (3)
Total oats (3)
Potatoes (3)
Winter rape (2)
Spring rape (2)
Total rape (3)
Sunflower (9)
Winter wheat (3)
Spring wheat (2)
Total wheat (2)
Wine (2)
Cluster code
1
2
3
4
5
6
7
8
9

(b) 1930-1959

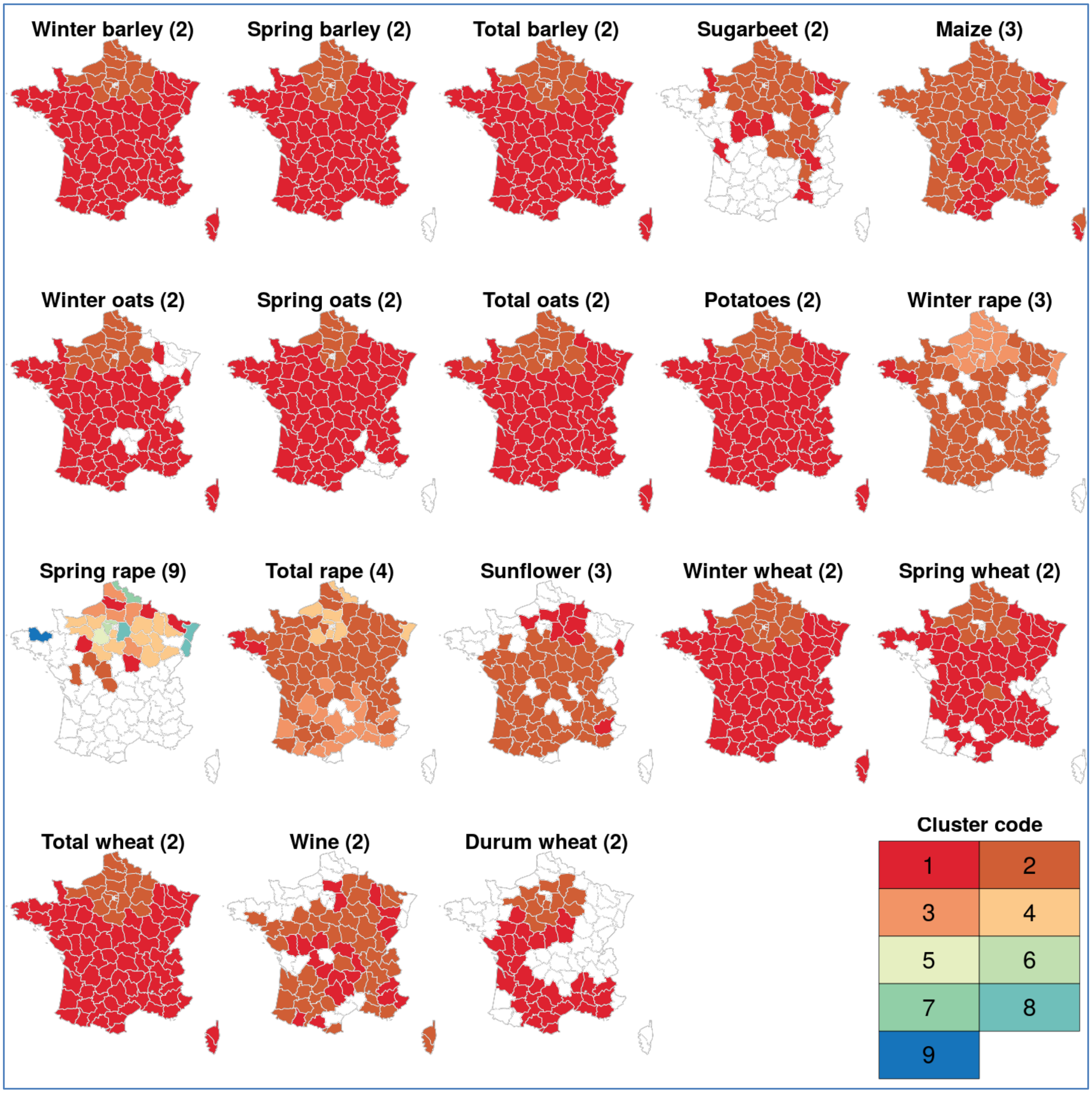
Winter barley (2)
Spring barley (2)
Total barley (2)
Sugarbeet (2)
Maize (3)
Winter oats (2)
Spring oats (2)
Total oats (2)
Potatoes (2)
Winter rape (3)
Spring rape (9)
Total rape (4)
Sunflower (3)
Winter wheat (2)
Spring wheat (2)
Total wheat (2)
Wine (2)
Durum wheat (2)
Cluster code
1
2
3
4
5
6
7
8
9

(c) 1960-1989

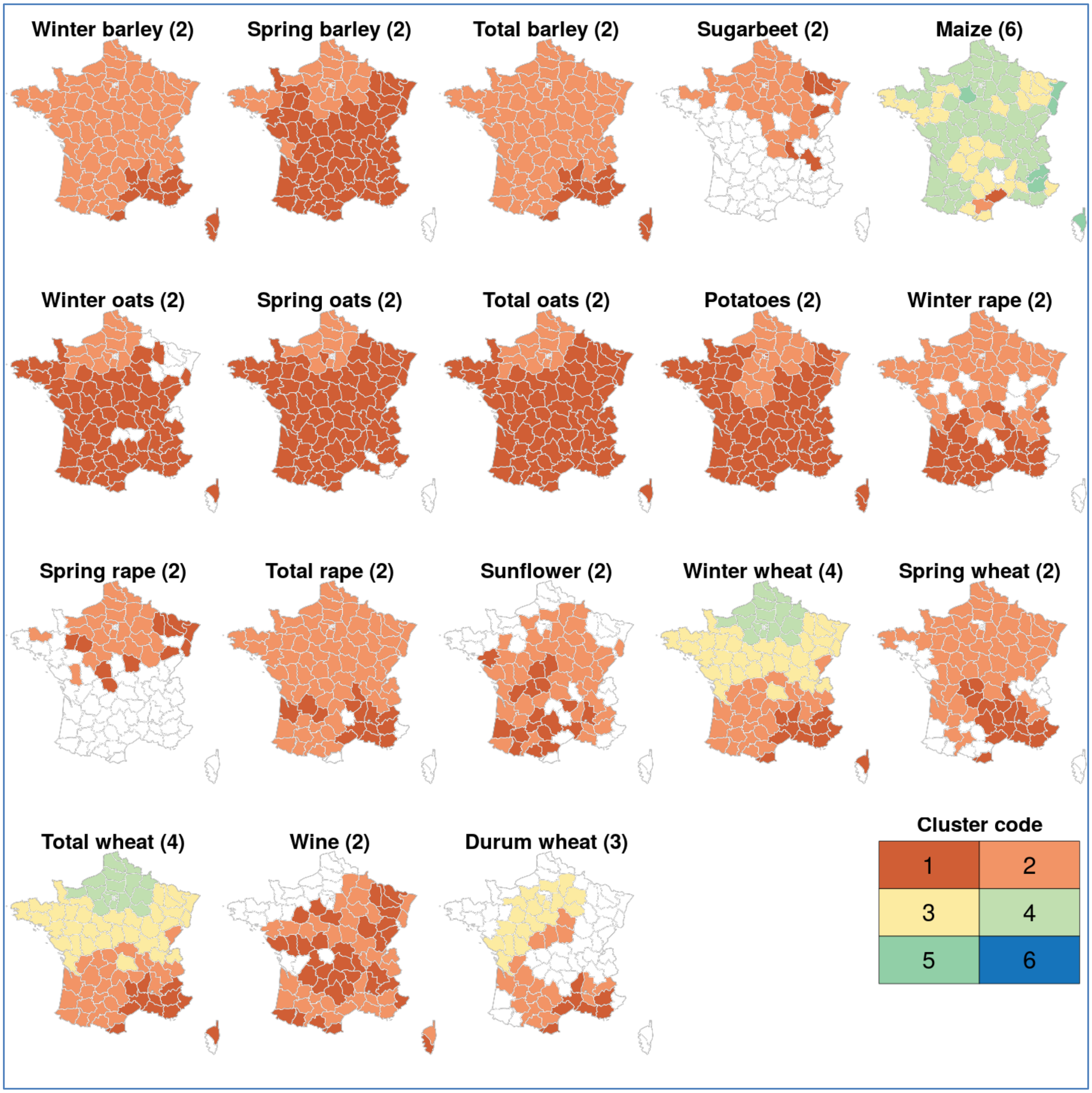

(d) 1990-2016

SI Figure 6: Spatial clustering of departments in different time frames. (a) 1900-1929, (b) 1930-1959 with two-season crops starting only in 1943, (c) 1960-1989, (d) 1990-2016

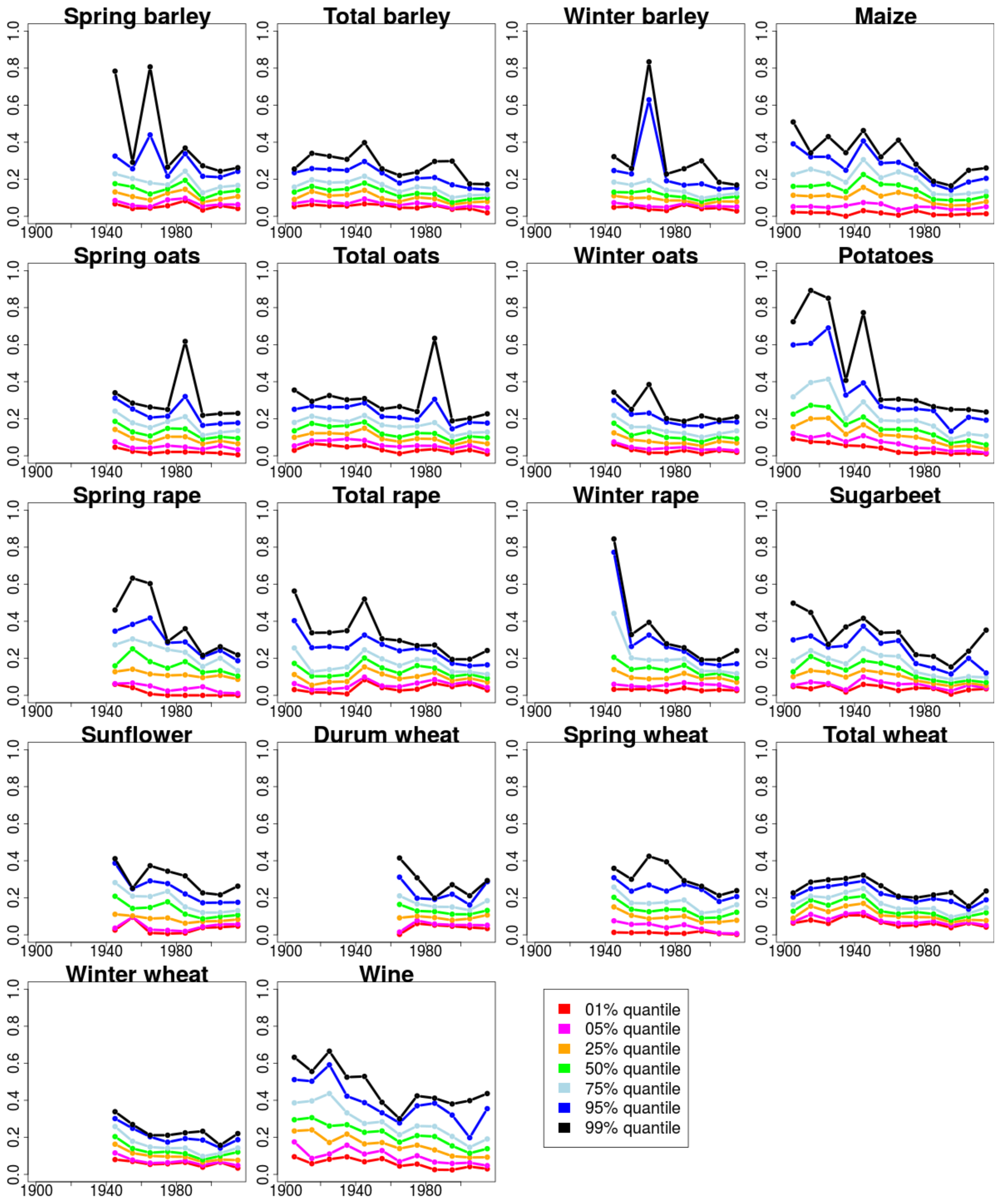

(a) Relative yield variation CV = Coefficient of Variation (unitless)

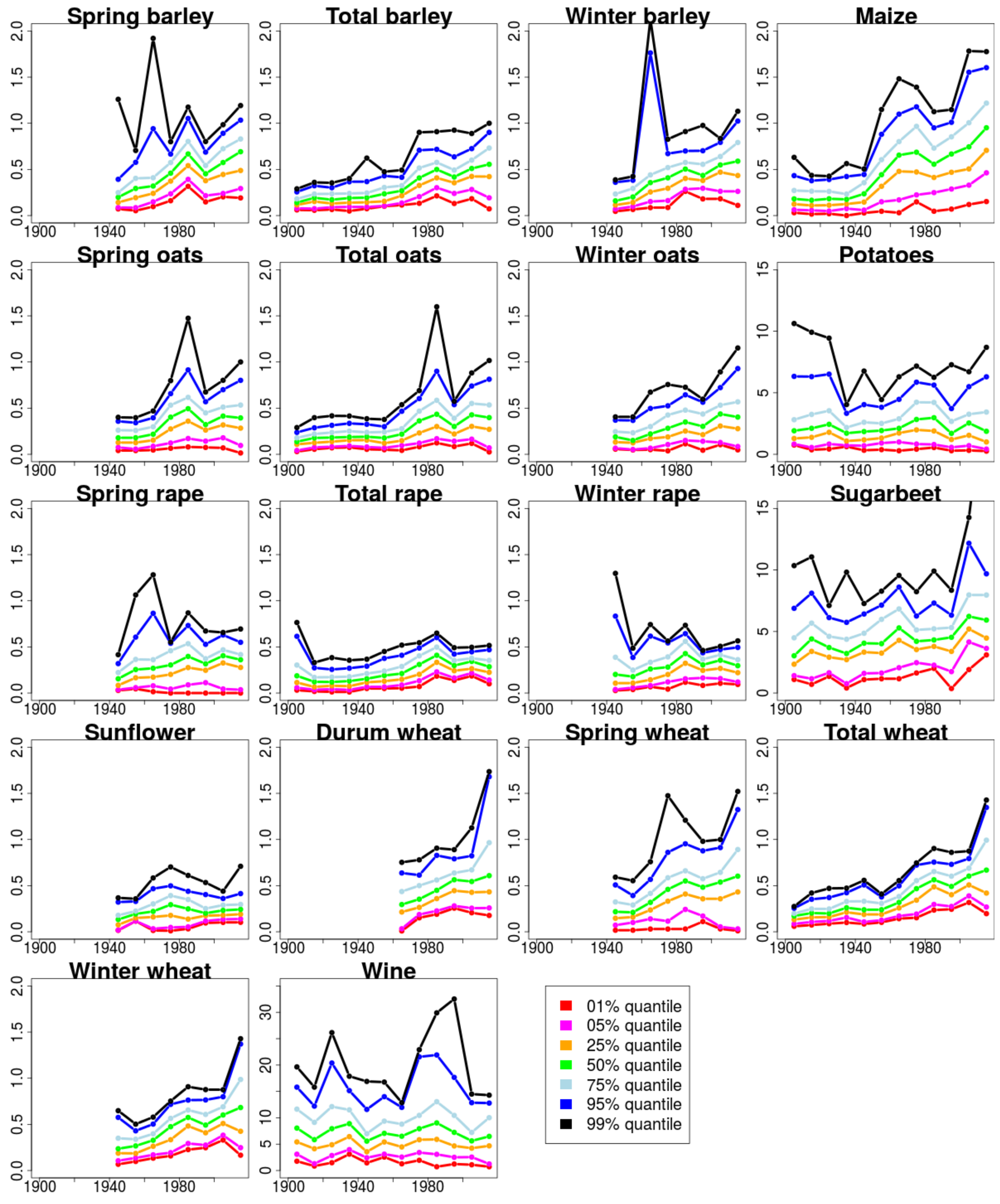

(b) Absolute yield variation (Standard deviation, in t/ha except for wine with hl/ha)

SI Figure 7: Trends in (a) relative and (b) absolute yield variability for all crop types.

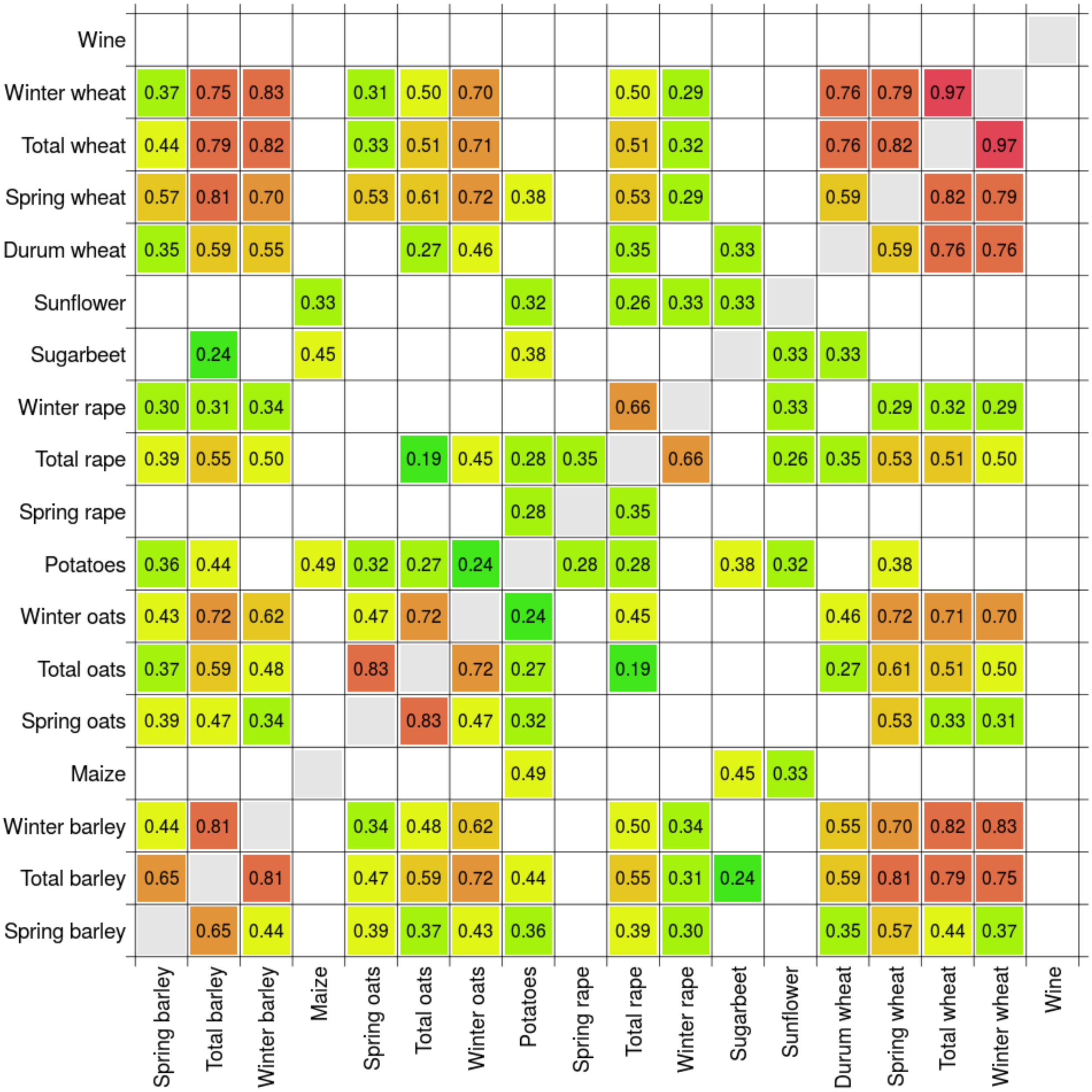

(a) 5% quantile of yields

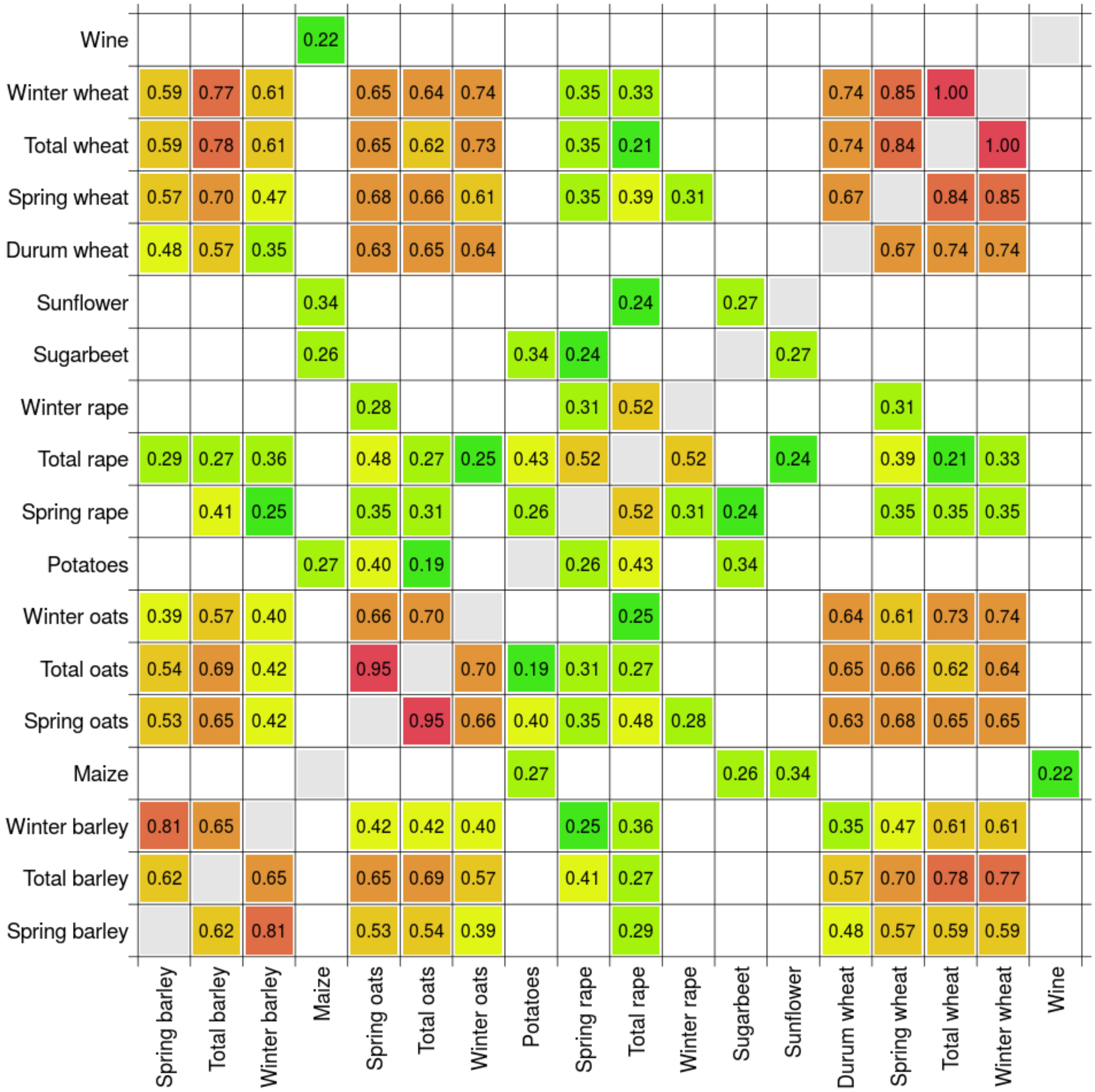

(b) 95% quantile of yields

SI Figure 8: Temporal correlation of yield residuals for national (a) 5% and (b) 95% quantiles (Pearson's r); only significant correlations are plotted ($p < 0.05$). Color coding represents correlation strength, from green = low to red = strong.

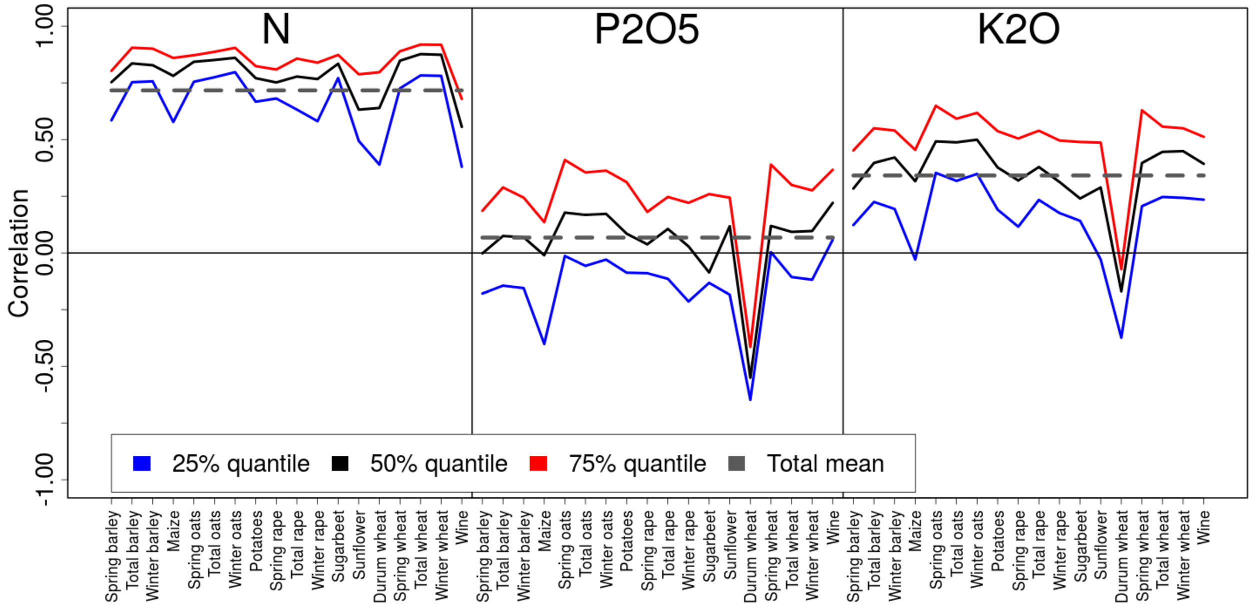

(a) Correlations between yield and fertilizer application trends

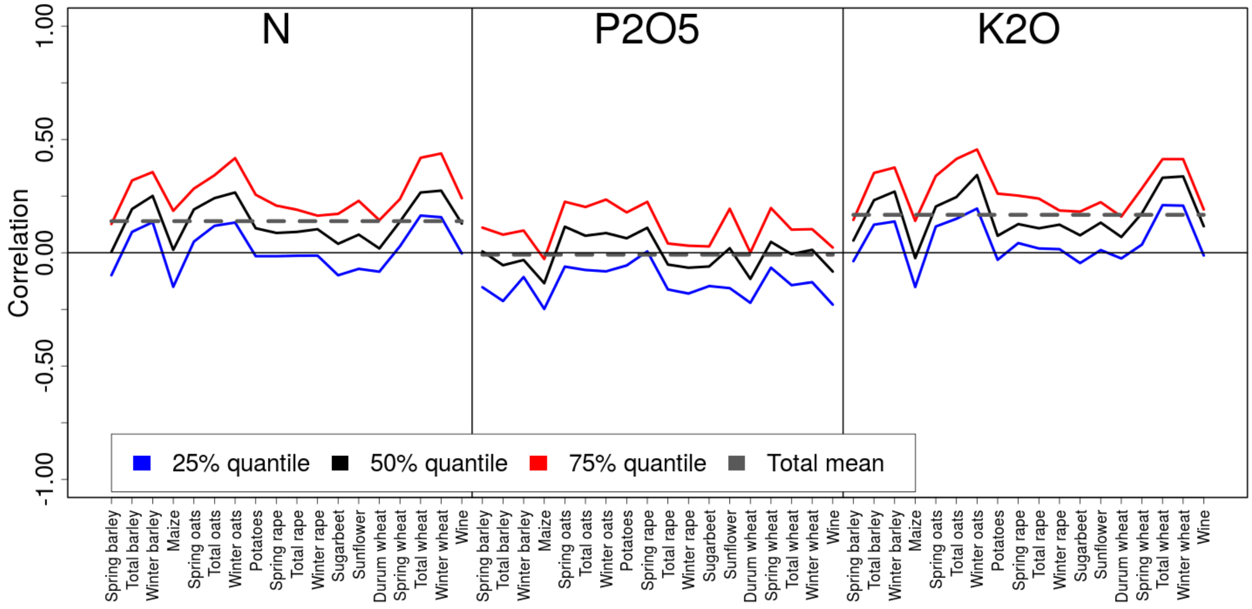

(b) Correlations between yield and fertilizer application residuals

SI Figure 9: Correlation of (a) yield trends with fertilizer application trends and (b) yield residuals with fertilizer application residuals. Fertilizer application data are not crop-specific.

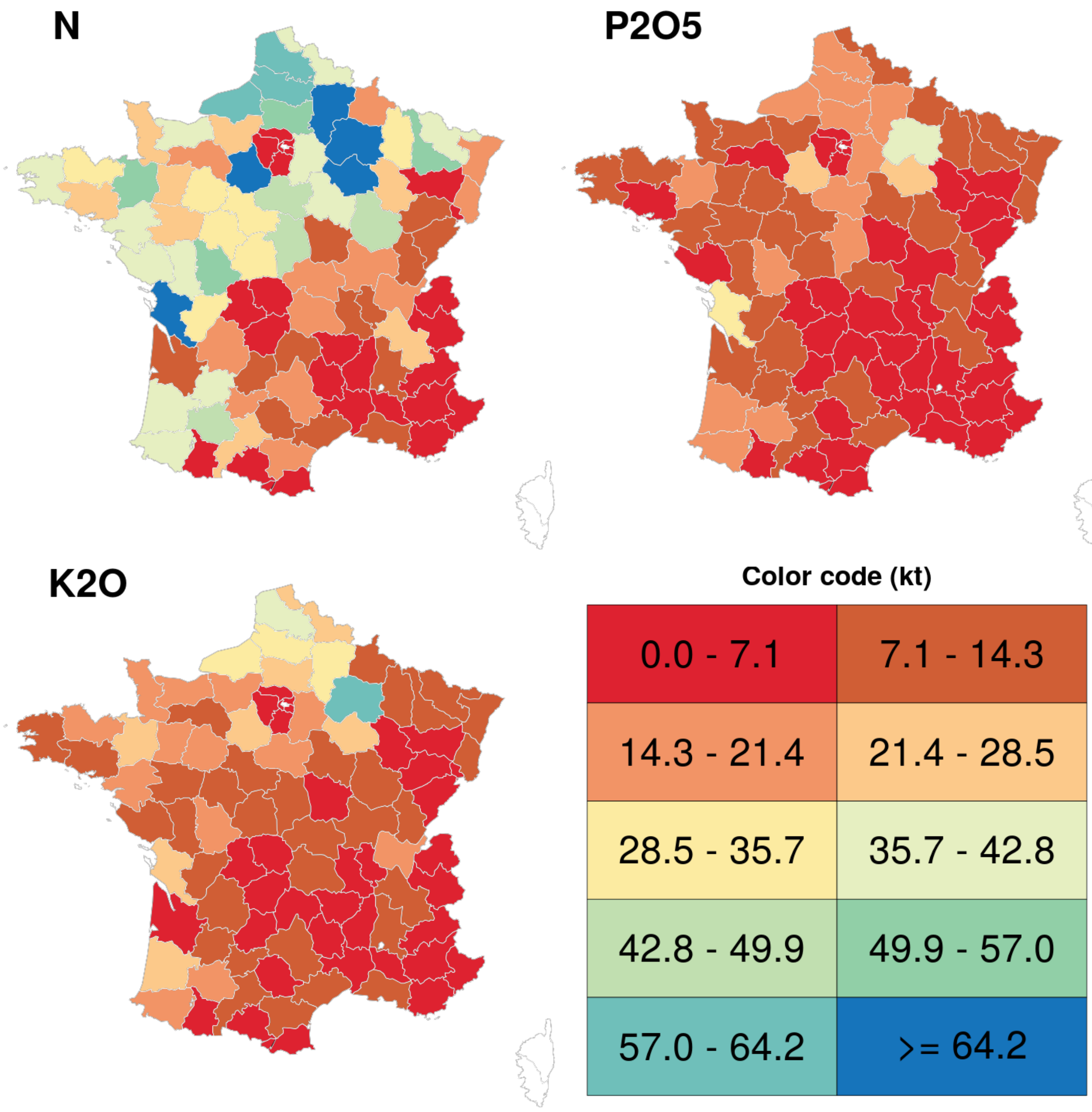

SI Figure 10: Fertilizer usage (kilotons, kt) per department, as average between 1990 and 2013. For Corsica data is only available until 1975.

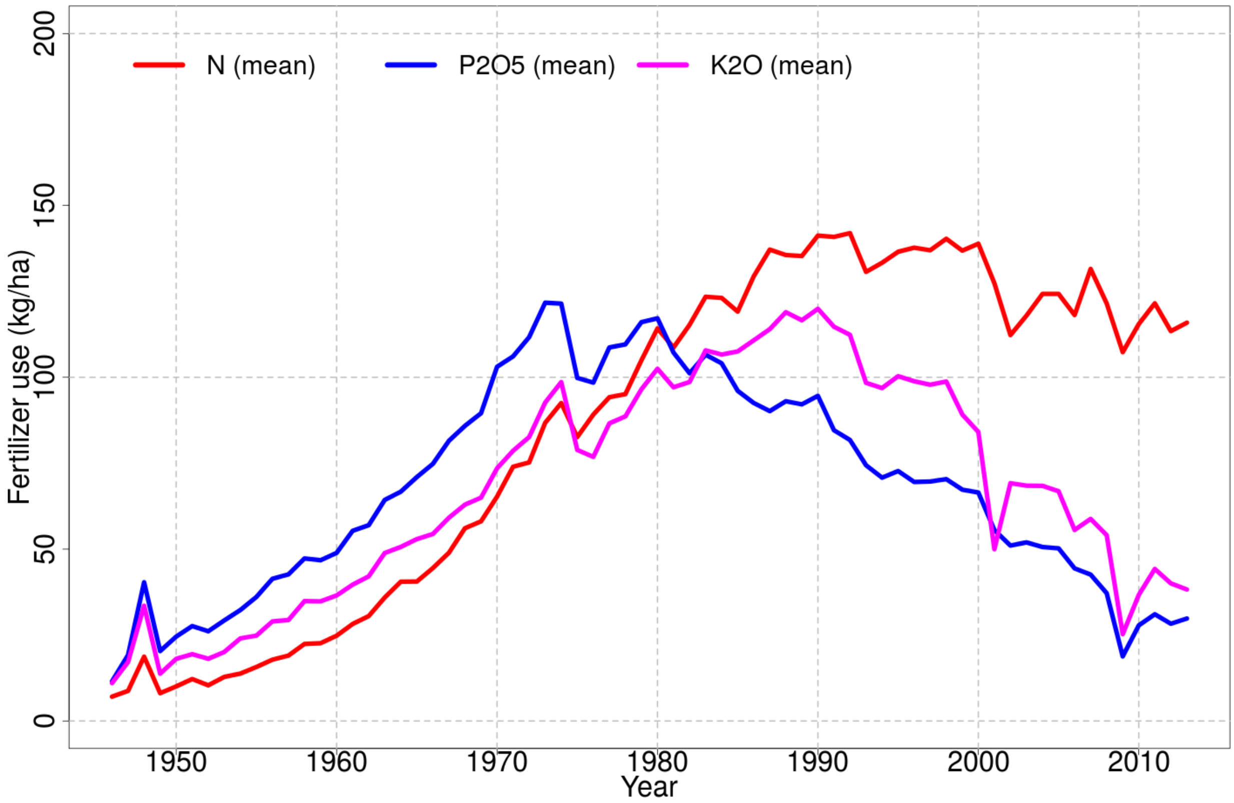

SI Figure 11: Trends in fertilizer application per area over time. Fertilizer usage is only provided per department and is divided by the total agricultural area for the ten crops analyzed here. The annual mean over all departments is shown.

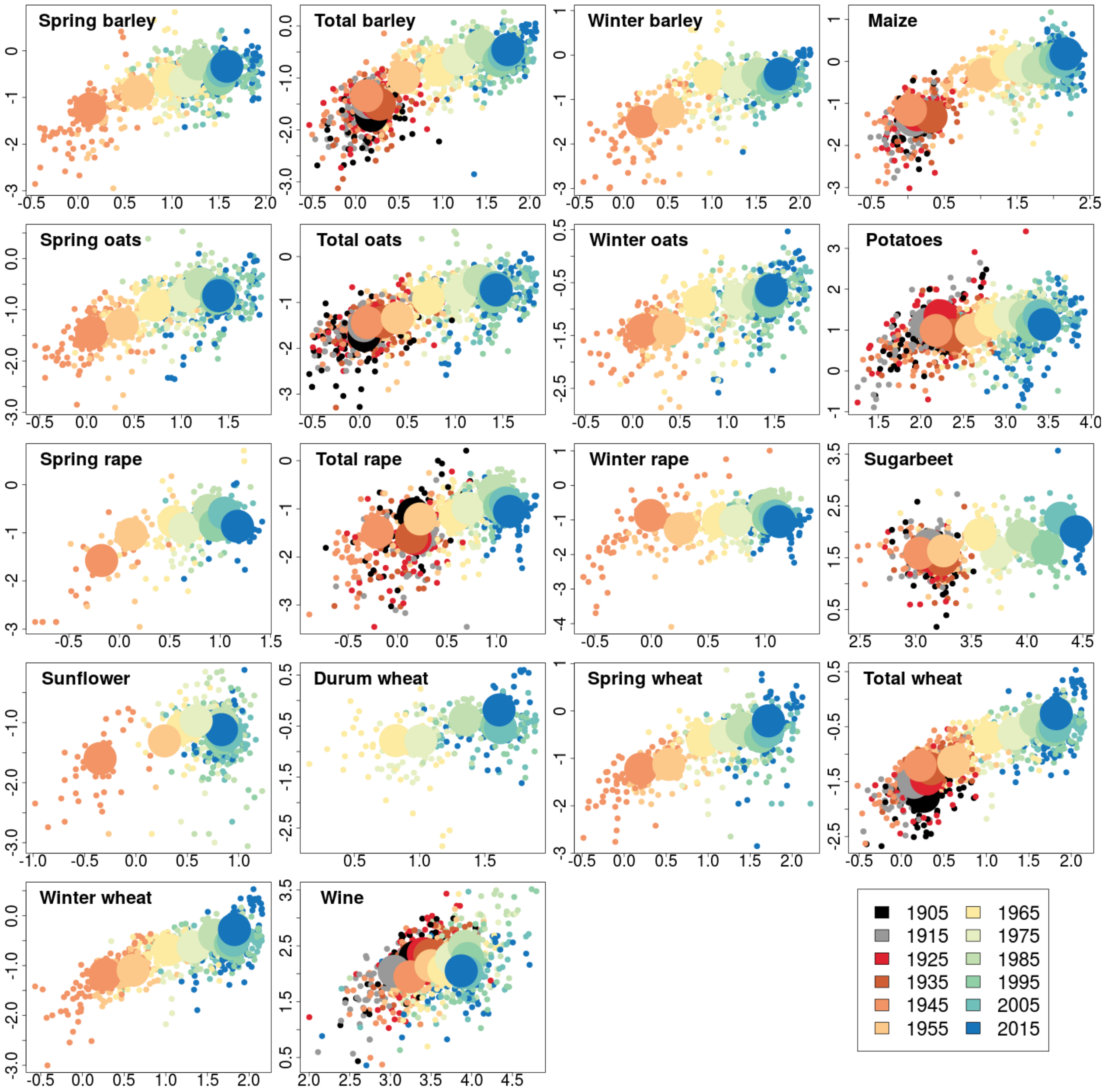

SI Figure 12: Taylor power plots for each crop over all decades. Log-transformed mean yields (x-axis; t/ha except for wine with hl/ha) are plotted against log-transformed standard deviations (y-axis). Different colors denote different decades. Each small dot corresponds to one department, while the large circles represent means across all departments for each decade. Entries with standard deviations smaller than 0.01 t/ha were removed before analysis to avoid large negative log values. Decades are calendar decades, i.e. the "1905" points are calculated from yields in 1900-1909. The "2015" results therefore contain only an incomplete decade, 2010-2016.

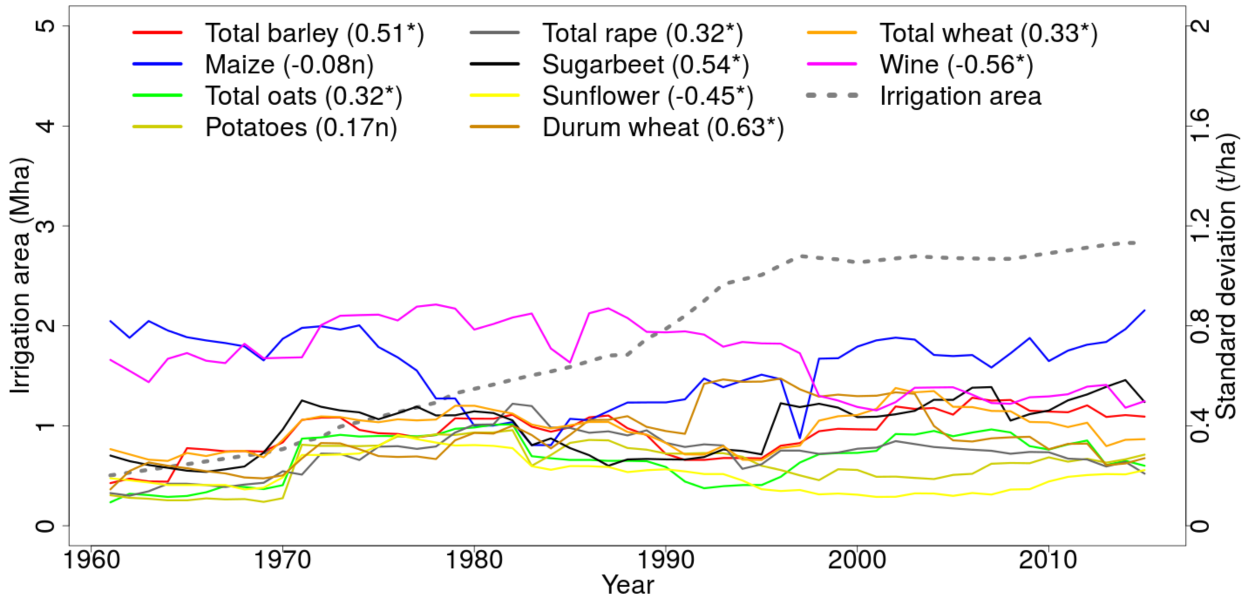

SI Figure 13: Time series of irrigated agricultural area on national level (in Mha, grey dashed line; left y axis; not crop-specific) by FAOStat and of moving standard deviations of yield residuals (window size of 11, with edge filling; colored lines; right y axis). Correlation strengths (Pearson's *r*) and their significance (n = not significant, * = significant at 0.05 level) are indicated behind each crop's name. Note that the standard deviations for potatoes, sugarbeet and wine have been scaled by 0.1 for display reasons, and the unit for wine is hl/ha (before scaling).

Supplementary Tables

SI Table 1: Data set description for yields on department level. A total of 11,232 data points per crop (96 departments in 117 years) would be possible.

**Crop (*French name*)**	**Seasonal type**	**Years with data**	**Filtered outliers (fraction of data)**	**Number of data points after filtering**
Barley (*Orge*)	Spring	1943-2016	271 (4.6%)	5,878
	Winter	1943-2016	170 (2.8%)	6,130
	Total	1900-2016	35 (0.3%)	10,561
Maize (*Maïs*)	(none)	1900-2016	63 (0.7%)	8,529
Oats (*Avoine*)	Spring	1943-2016	23 (0.4%)	6,146
	Winter	1943-2016	13 (0.2%)	5,737
	Total	1900-2016	25 (0.2%)	10,555
Potatoes (*Pommes de terre*)	(none)	1900-2016	292 (2.8%)	10,253
Rape (*Colza*)	Spring	1943-2016	204 (8.7%)	2,354
	Winter	1944-2016	711 (14.5%)	4,887
	Total	1900-2016	165 (2.1%)	7,768
Sugar beet (*Betterave*)	(none)	1900-2016	53 (1.1%)	4,831
Sunflower (*Tournesol*)	(none)	1944-2016	60 (1.7%)	3,481
Soft wheat (*Froment, Blé tendre*)	Spring	1943-2016	19 (0.4%)	4,917
	Winter	1943-2016	31 (0.5%)	6,784
	Total	1900-2016	8 (0.1%)	10,618
Durum wheat (*Blé dur*)	Total	1961-2016	4 (0.2%)	2,615
Wine (*Vigne*)	(none)	1900-2016	222 (2.6%)	8,558
**Total yield data points**			**2,369 (2.0%)**	**120,602**

SI Table 2: Mapping of cluster codes (Figure 3 in main paper) to mean yields (in t/ha)

Crop	Time frame	Cluster code	Mean yield
Spring barley	1930-59	1	1.1
		2	1.7
	1960-89	1	2.9
		2	3.8
	1990-2016	1	4
		2	6.3
Maize	1900-29	1	0.9
		2	1.2
	1930-59	1	1
		2	1.4
	1960-89	1	3.8
		2	4.9
		3	5.9
	1990-2016	1	4.2
		2	4.9
		3	7.5
		4	8.6
		5	9.8
Potatoes	1900-29	1	5.1
		2	8.5
		3	10.9
	1930-59	1	8
		2	12.1
		3	19
	1960-89	1	18.9
		2	29.5
	1990-2016	1	26.1
		2	40.7
Winter wheat	1930-59	1	1.1
		2	1.7
		3	2.2
	1960-89	1	3.5
		2	5
	1990-2016	1	3.5
		2	5.3
		3	6.7
		4	8.1
